# Mechanisms of experience-dependent place-cell referencing in hippocampal area CA1

**DOI:** 10.1038/s41593-025-01930-5

**Published:** 2025-04-01

**Authors:** Fish Kunxun Qian, Yiding Li, Jeffrey C. Magee

**Affiliations:** 1 Department of Neuroscience, Baylor College of Medicine, Houston, TX USA; 2 Howard Hughes Medical Institute, Baylor College of Medicine, Houston, TX USA

**Keywords:** Long-term potentiation, Hippocampus, Reward

## Abstract

Hippocampal CA1 place cells (PCs) encode both space- and goal-referenced information to support a cognitive map. The mechanism of this referencing and the role of experience remain poorly understood. Here we longitudinally recorded PC activity while head-fixed mice performed a spatial learning task on a treadmill. In a familiar environment, the CA1 representation consisted of PCs that were referenced to either specific spatial locations or a reward goal in approximately equal proportions; however, the CA1 representation became predominately goal-referenced upon exposure to a novel environment, as space-referenced PCs adaptively switched reference frames. Intracellular membrane potential recordings revealed that individual CA1 neurons simultaneously received both space- and goal-referenced synaptic inputs, and the ratio of these inputs was correlated with individual PC referencing. Furthermore, behavioral timescale synaptic plasticity shaped PC referencing. Together, these results suggest that experience-dependent adjustment of synaptic input shapes PC referencing to support a flexible cognitive map.

## Abstract (web summary)

Through multiday imaging and acute whole-cell recordings in behaving mice, Qian, Li and Magee provide insight into place field formation in general and specifically how the hippocampus adaptively remaps for flexible goal-directed navigation.

## Main

Goal-directed spatial navigation in a rapidly changing environment requires neuronal mechanisms that flexibly use both space- and goal-related information^1,2^. Central to the mammalian navigation system are hippocampal place cells (PCs) that robustly increase their firing rates when an animal enters a specific place (place field (PF)) in the environment^3^, with the population of PCs producing an accurate representation of the animal’s location in the environment^4^. Notably, the firing of PCs is relatively stable across sessions^5^ and strongly supports an animal’s flexible behavioral choices^6–9^. Thus, the collective activity of PCs is commonly believed to serve as a neural substrate for a cognitive map of space in the brain^1,2,10–13^.

In freely behaving animals, PCs are thought to be primarily controlled by, and therefore referenced to, a constellation of salient environmental spatial features (for example, visual landmarks)^14–16^. Modifications of these features often cause PCs to alter their PFs (remapping), leading to distinct representations for different environments^15,17,18^. PCs within a single environment can coherently remap in response to environmental feature modifications and are therefore hypothesized to anchor to a specific spatial reference frame^1,6,18–20^. Within this framework, medial entorhinal cortex (MEC) is proposed to support hippocampal neuronal dynamics by providing a universal path integration-based activity updating mechanism^10,21–24^.

In contrast to the above view of a single, dominant reference frame governing the hippocampal map, evidence is accumulating that hippocampal representations may integrate multiple reference frames^2,7–9,25–33^. For instance, cue manipulation can cause local remapping, affecting only the PFs near the altered cue^14,15^. Further, a series of elegant cue-mismatch studies revealed heterogeneous responses of different subsets of PCs within the same environmental context^34–40^. Finally, the lateral entorhinal cortex (LEC) provides reward-referenced spatial inputs that are distinct from MEC^41,42^. Together, the hippocampus might be well suited for integrating multiple reference frames to produce a conjunctive cognitive map of space^11,43^.

Although previous studies have shown that in a randomly foraging task, PCs are flexibly referenced to different cues depending on previous experiences^44–46^, how general the phenomenon of flexible hippocampal PC referencing is, and its cellular-level mechanisms remain unknown. To examine this, we sought to specifically determine the reference frame of individual PCs in a CA1 representation of the environment during a goal-directed spatial learning task, the degree to which this referencing is plastic and its synaptic mechanisms.

## Results

**Fig. 1:**
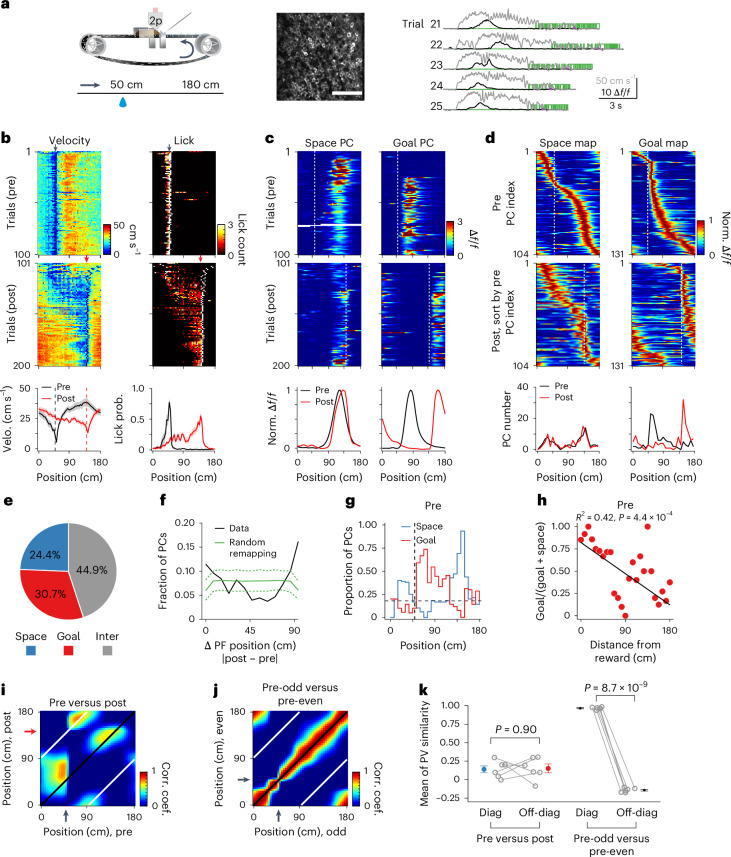
Balanced goal- and space-referenced spatial coding in a familiar environment. **a**, A mouse on a treadmill for a water reward at a fixed location (left). Field of view showing GCaMP6f-expressing pyramidal neurons in the dorsal CA1 (middle). Scale bar, 100 μm. Traces of Δ*f*/*f* from a PC (black), running velocity (gray) and licks (green) (right). Purple dots indicate the reward locations. **b**, Running (left) and licking (right) behavior of a representative animal on a familiar belt before (pre; black arrow indicates the reward at 50 cm) and after (post; red arrow indicates the reward at 140 cm) a reward switch (top/middle). Mean velocity (velo., left) and licking probability (prob., right) of all mice (*n* = 6) (bottom). Shading indicates s.e.m. **c**, One example space (left) and goal PC (right) illustrating spatial Δ*f*/*f* across laps (pre, top; post, middle). Peak-normalized (norm.) mean Δ*f*/*f* across space (bottom). **d**, PC activity on a familiar belt. Peak-normalized mean Δ*f*/*f* of all space (left) and goal PCs (right) in pre and post (top/middle). Maps are sorted by their peak locations in pre. PC numbers across space in pre and post (bottom). **e**, Fraction of different PC profiles (*n* = 427 PCs, 6 mice). **f**, PC fractions across PF shift distance. Green dashed lines show the top/bottom fifth percentile of simulated values (random remapping model). **g**, Proportion of space and goal PCs across space in pre. Horizontal dashed line: expected fraction (~0.167, random remapping). **h**, Ratio of goal PC to the sum of goal and space PCs across distance from reward (pre). Black line shows weighted two-sided linear fit. **i**, Correlation matrix showing Pearson’s correlation coefficient (corr. coef.) between PVs at all locations from pre and post. Black and white lines indicate diagonal and off-diagonal, respectively. **j**, Same as **i**, but between PVs from odd and even trials in pre. **k**, Mean Pearson’s correlation coefficient along the diagonal (diag) and off-diagonal (off-diag) axis (*n* = 6 mice for all conditions). Pre versus post: 0.14 ± 0.046 versus 0.15 ± 0.060; *P* = 0.90; pre-odd versus pre-even: 0.97 ± 0.0065 versus −0.14 ± 0.012, *P* = 8.7 × 10^−9^. Paired two-sample two-sided *t*-test for both comparisons. Data are mean ± s.e.m. Vertical dashed lines indicate reward locations (**b**–**d**,**g**). Source data

**Extended Data Fig. 1:**
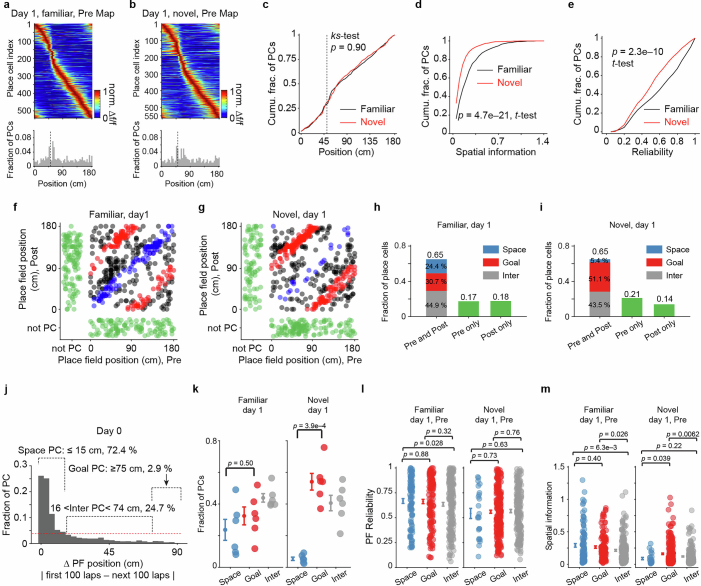
CA1 PC representation before and after a reward switch. **a**, Top: Peak-normalized Δf/f for PCs sorted by PF peak locations (familiar, Day 1, Pre, 6 mice). Bottom: PF peak distribution. Black dashed line: reward location. **b**, Same as **a** but for a novel belt (6 mice). **c–e**, Cumulative PC fractions across PF locations (**c**, *p* = 0.90, two-sample two-sided Kolmogorov–Smirnov test), spatial information (**d**, Familiar versus Novel, 0.23 ± 0.0093 versus 0.12 ± 0.0057, *p* = 4.7e–21), and PF reliability (**e**, Familiar versus Novel: 0.61 ± 0.01 versus 0.52 ± 0.01, *p* = 2.3e–10). **f**, PF position distribution (Post versus Pre, familiar, Day 1, 6 mice). Space, Goal, and Inter PCs: blue, red, and black. Green: Cells with PFs in Pre (114 cells) or Post only (116 cells). **g**, Same as **f** but for a novel belt (6 mice; 138 Pre-only, 91 Post-only cells). **h/i**, PC fractions for familiar (**h**) and novel (**i**) belts (6 mice each). **j**, PC classification criterion using PF position change (first versus second 100 laps, familiar; Day 0: Last day with fixed reward, 1737 cells, 14 mice). Last bin above chance: Bin #5 (15 cm). Criterion: ≤15 cm, 16–74 cm, and ≥75 cm for Space, Goal, and Inter PCs. **k**, Fraction of Space, Goal, and Inter PCs for familiar (23.7% ± 6.7%, 32.5% ±5.6%, 43.9% ± 2.7%) and novel belts (5.3% ± 1.1%, 54.1% ± 5.3%, 40.6% ± 4.9%). Each dot: one mouse. 6 mice each. Paired two-sided *t*-test. **l**, PF reliability for Space, Goal, and Inter PCs for familiar (0.67 ± 0.02, 0.66 ± 0.02, 0.64 ± 0.02) and novel belts (0.54 ± 0.05, 0.56 ± 0.02, 0.57 ± 0.02). **m**, Spatial information for Space, Goal, and Inter PCs on familiar (0.29 ± 0.027, 0.26 ± 0.018, 0.21 ± 0.014) and novel belts (0.087 ± 0.018, 0.16 ± 0.011, 0.12 ± 0.0091). Unpaired two-sided *t*-test for **d**–**e** and **l**–**m**. For **f**–**g**, and **l–m**: 104 Space, 131 Goal, and 192 Inter PCs (familiar, 6 mice); 23 Space, 217 Goal, and 185 Inter PCs (novel, 6 mice). For panels **k**–**m:** The exact *p*-values are presented in figures and data are presented as mean ± SEM. Source data

Here we recorded from adult head-fixed mice engaged in a hippocampal-dependent goal-directed spatial learning task on a linear treadmill^47,48^. The task required head-fixed mice to run for a sucrose water reward (delivered at 50 cm) on a 180-cm-long track (belt A), uniformly covered with distinct tactile cues to aid navigation (Fig. 1a). After training, mice typically developed anticipatory slowing and licking before the reward delivery site, indicating that the location of the reward had been successfully learned (Fig. 1b, ‘pre’, before the reward switch). We performed in vivo two-photon calcium imaging to optically record populations of PC activity from CA1 pyramidal neurons that transgenically expressed GCaMP6f (Fig. 1a). Consistent with previous studies, we observed large numbers of PCs per animal (90.2 ± 14.2 PCs per animal, *n* = 6 mice) that exhibited reliable trial-by-trial fluorescence increases at selective locations^18,31,48,49^ (Fig. 1c). Further, these PCs tiled the entire track, with an elevated density found around the reward location^30,31,48,50–52^ (over-representation; Extended Data Fig. 1a).

### Balanced space- and goal-referenced spatial coding in a familiar environment

**Extended Data Fig. 2:**
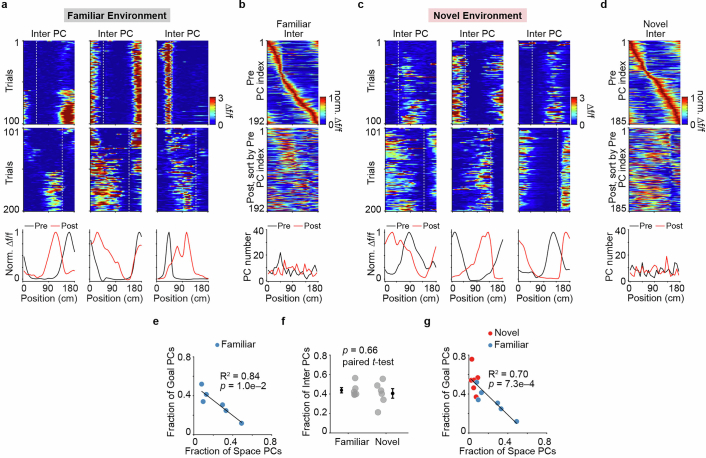
Intermediate PCs from day 1 on a familiar versus novel belt. **a**, Three example Inter PCs. Colormaps show spatial Δf/f across laps during Pre and Post. White dashed lines mark reward locations. Bottom: Peak-normalized mean Δf/f. **b**, PC representation. Top and Middle: Peak-normalized mean Δf/f for all Inter PCs (Pre and Post; n = 192 Inter PCs from 6 mice). Maps are sorted by their peak locations in Pre. Bottom: PC numbers across space in Pre and Post. **c–d**, Same as **a–b** but for a novel belt (n = 185 Inter PCs from 6 mice). **e**, The fraction of Goal versus Space PCs (Familiar belt). The black line is a two-sided linear fit. Each dot represents one mouse. n = 6 mice. **f**, The fraction of Inter PCs (familiar versus novel, 43.9 % ± 2.7 % versus 40.6 % ± 4.9 %, respectively). *p* = 0.66, paired two-sided *t*-test. Each dot represents one mouse. n = 6 mice for both conditions. **g**, Same as **e** but including both familiar and novel belts. n = 12 mice (6 from familiar, 6 from novel). Each dot represents one animal. Black line is a two-sided linear fit. Data are presented in mean ± SEM. Source data

Determining the contribution of distinct reference frames to PC activity requires independent experimental manipulations of each reference frame. In our spatial learning task, distinct tactile cues on the belt potentially provide spatial reference points^49,51^ while a mouse’s stereotypical running pattern between reward goals provides a goal reference, where integration of self-motion over time is, theoretically, sufficient for the animal to update its position^21,23,53^. To investigate whether the CA1 PC representation references to reward goal- or spatial-cue-related reference frames in our task, we switched the reward from 50 cm to 140 cm (half of the belt length) after the first 100 trials. This manipulation dissociates the animal’s reward goal-related and spatial cue-related reference frames, allowing us to study their respective influences on each PC’s activity. As the mice adapted their anticipatory slowing and licking to the new reward location (Fig. 1b, ‘post’, after the reward switch), we observed dramatic PC remapping in CA1 (refs. ^15,18,31^) such that PCs changed their PF locations, pre-existing PCs disappeared and new PCs emerged (Extended Data Fig. 1f). Of those PCs with a reliable PF both before and after the reward switch (65% of all PCs, *n* = 6 mice; Extended Data Fig. 1h), we identified 24.4% as spatial-cue-referenced (‘space-referenced’) PCs that maintained a stable PF relative to the spatial cues after the reward switch (PFs shifted <15 cm; Fig. 1c–e and Extended Data Fig. 1j), 30.7% as reward-goal-referenced (‘goal-referenced’) PCs that shifted their PFs in space but maintained the same relation to the reward on each lap (PFs shifted between 75 and 90 cm; Fig. 1c–e), and a remaining 44.9% that were intermediate because their PFs shifted between 16 and 74 cm (Fig. 1e and Extended Data Figs. 1k and 2a,b). Moreover, space- and goal-referenced PCs had similar PF reliability and spatial information (Extended Data Fig. 1l,m). The population distribution of PF displacement displayed two peaks toward 0 and 90 cm, indicating that PC remapping in CA1 was not random, but instead consisted of PCs that were primarily either space- or goal-referenced (Fig. 1f). In addition, the fraction of space-referenced PCs had a strong inverse correlation with the fraction of goal-referenced PCs (Extended Data Fig. 2e), indicating that these two reference frames might compete with each other^34^.

**Extended Data Fig. 3:**
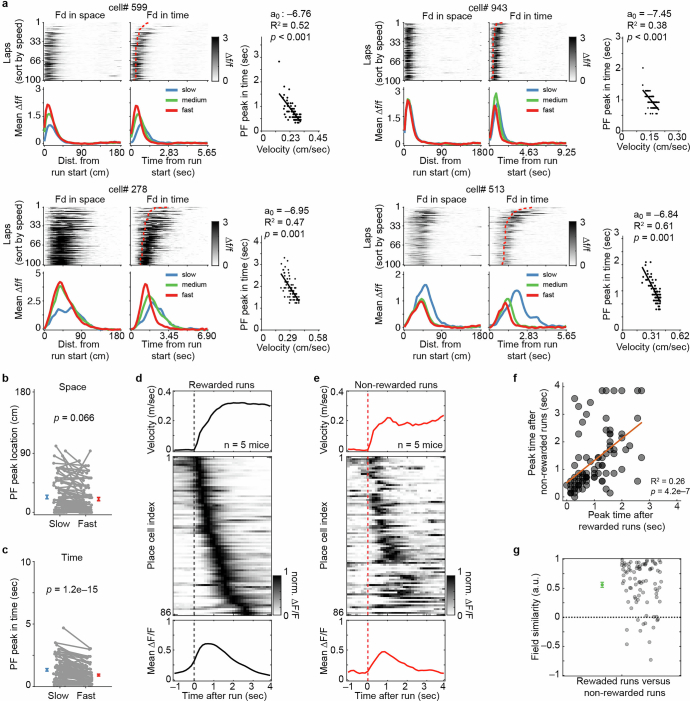
Goal PCs encoded reliable distance but not time information after the running start and didn’t require reward per se. **a**, Four example Goal PCs showing relatively consistent spatial but not temporal coding at varying running speeds. The heatmaps show Δf/f values for space and time across laps, sorted by the running speed from the running onset to the peak PF location. Group-averaged Δf/f traces in red, green, and blue traces represent fast, medium, and slow speeds, respectively. Red dashed line: Time after running onset to the PF peak. The across-lap PF peak timing against running velocity is also plotted. The black line: Two-sided linear fit. **b**, PF peak position in space during slow (26.1 ± 2.6 cm) versus fast (23.0 ± 2.3 cm) runs. *p* = 0.066. **c**, PF peak position in time during slow (1.3 ± 0.10 s) versus fast runs (0.91 ± 0.075 s). *p* = 1.2e–15; one-sample two-sided Kolmogorov–Smirnov test for panels **b–c**. For panels **a**–**c**, only Goal PCs with PFs within 90 cm after the running onset were included (n = 90 Goal PCs from 6 mice, Pre, familiar). **d**, Top: Mean running velocity around the start of rewarded runs. Middle: Peak-normalized mean activity (Δf/f) in time around the start of rewarded runs for Goal PCs (Familiar, Day 1, Pre) sorted by the peak timing. Goal PCs with PFs located more than 90 cm after the reward were excluded. n = 86 Goal PCs. Bottom: Population mean Δf/f in time. **e**, Same as **d**, but for non-rewarded runs. PCs are sorted by peak timing during rewarded runs. **f**, Comparison of the activity peak timing between rewarded and non-rewarded runs. Each dot indicates one neuron. Orange line: Two-sided linear fit. **g**, PC field similarity between rewarded and non-rewarded runs (0.56 ± 0.043; *p* = 8.9e–22, paired two-sided *t*-test). Each black dot represents one neuron. Horizontal dashed line marks 0. For panels **d–g**: n = 86 neurons from 5 mice. One mouse did not exhibit non-rewarded runs, resulting in 43 non-rewarded running episodes from 5 mice. Data: Mean ± SEM. Source data

Previous studies suggested that spatial coding using path integration degrades progressively as the animal traverses away from its original reference point, while spatial sensory cues, which are fixed in external space, can correct errors accumulated during navigation^21,23,53^. We therefore examined the spatial distribution of goal- and space-referenced PCs and found that goal-referenced PCs preferentially clustered near the onset of running after the reward (peak at 60 cm, that is, 10 cm after the reward site; Fig. 1d,g), whereas space-referenced PCs were predominant over the rest of the track (peaks at 144 cm and 36 cm; Fig. 1d,g), consistent with previous studies^34^. Notably, more than half of the goal-referenced PCs had PFs located 30 cm distant in space (median, 32 cm; Fig. 1d), and all were many seconds in time from the reward site (time between reward delivery and the start of running: 6.8 ± 0.18 s, *n* = 6 mice, first 100 laps before the reward switch), indicating that they did not simply represent appetitive reward responses^31,54^. Furthermore, goal-referenced PCs that clustered after the reward (<90 cm) demonstrated relatively stable PF positions in space relative to the start of running across both slow and fast running laps (mean PF position in space: 26.1 ± 2.6 versus 23.0 ± 2.3 cm, slow versus fast laps, respectively; *P* = 0.066; Extended Data Fig. 3a,b). In contrast, the activity of these PCs peaked later in time during slower runs compared to faster ones (mean PF position in time, 1.3 ± 0.10 versus 0.91 ± 0.075 s, slow versus fast laps, respectively; *P* = 1.2 × 10^−15^; Extended Data Fig. 3a,c), indicating that it is unlikely these goal-referenced PCs were determined solely by a strictly time-dependent process after running^55^. At further distances from the reward site, the proportion of goal- versus space-referenced PCs decreased (Fig. 1h). Notably, similar sequential activity was present after the start of running at non-rewarded locations (Extended Data Fig. 3d–g). Thus, reward per se is not required to elicit appropriate sequential activity for the goal-referenced PCs^56,57^.

To examine how PC remapping influenced ensemble spatial coding before and after the reward switch, we performed a population vector (PV) analysis. In the correlation matrix formed from the activity before and after the reward switch, the single region of high correlation along the off-diagonal (white line, Fig. 1i) corresponded to goal-referenced PCs that primarily clustered at 60 cm (~10 cm after the reward site; Fig. 1d,g,i), suggesting that goal referencing primarily affects the beginning of the running episodes^32,34^. In contrast, two regions of high correlation along the diagonal (black line, Fig. 1i) corresponded to the space-referenced PCs that clustered at ~30 cm and ~140 cm (Fig. 1d,g,i), reflecting the predominance of space-referenced PCs in coding these positions relative to the spatial cues on the belt. This can be compared to the trial-by-trial correlations which show a single high correlation value around the diagonal (Fig. 1j,k). The mean of the PV correlation along the diagonal was nearly identical to that of the off-diagonal, suggesting that space- and goal-related reference frames have similar contributions to the ensemble spatial coding of the familiar environment (Fig. 1k). These results demonstrate that in a familiar environment both space- and goal-related reference frames exist supporting a coherent spatial representation in CA1.

### Experience alters the referencing of the CA1 PC representation

**Fig. 2:**
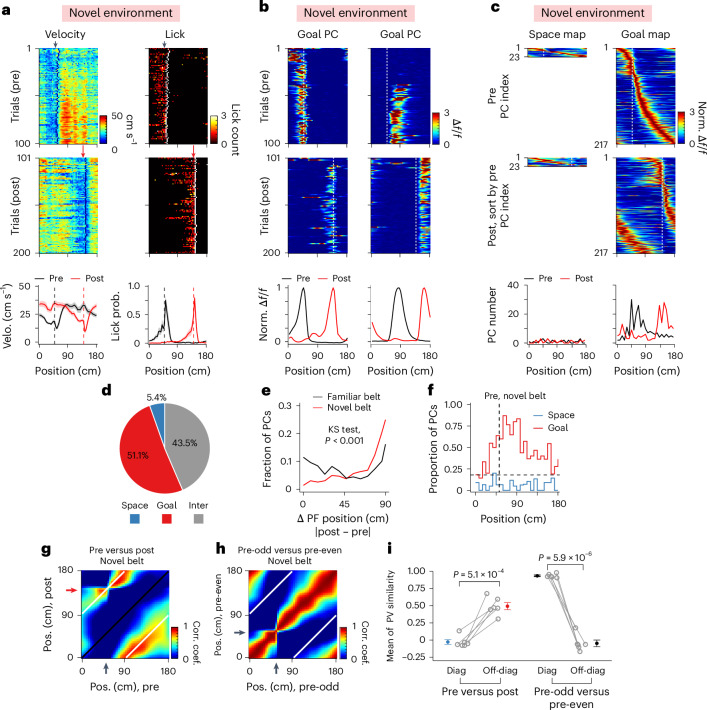
Predominant goal-referenced spatial coding in a novel environment. **a**, Running (left) and licking (right) behavior of an example animal on a novel belt before (pre; black arrow depicts reward at 50 cm) and after (post; red arrow depicts reward at 140 cm) a reward switch (top and middle). Mean velocity (left) and licking probability (right) for all mice (*n* = 6) (bottom). Shading indicates s.e.m. **b**, Spatial Δ*f*/*f* activity across laps during pre (top) and post (middle) from two goal PCs on a familiar belt: one with a PF before and the other after the reward. Vertical dashed white lines indicate reward locations. Peak-normalized mean Δ*f*/*f* across space in pre and post (bottom). **c**, PC Δ*f*/*f* activity on a novel belt. Peak-normalized mean Δ*f*/*f* of all space (left) and goal PCs (right) in pre and post (top and middle). Maps are sorted by their peak locations in pre. PC numbers across space in pre and post (bottom). **d**, The fraction of different PC profiles (*n* = 425 PCs from 6 mice). **e**, PC fractions across PF shift distance on familiar (black, *n* = 6 mice) and novel (red, *n* = 6 mice) belts (*P* = 1.8 × 10^−17^, two-sample two-sided Kolmogorov–Smirnov (KS) test). **f**, Proportion of space and goal PCs across space. The vertical dashed line indicates the reward location. The horizontal dashed line indicates the expected fraction (~0.167, random remapping). **g**, The Pearson’s correlation matrix between PVs at all locations from pre and post on a novel belt. Black and white lines indicate the diagonal and off-diagonal. **h**, Same as **g**, but for PVs from odd and even laps in pre. Pos., position. **i**, Mean PV correlation along the diagonal and off-diagonal on the novel belt (*n* = 6 mice for all conditions; pre versus post, −0.028 ± 0.033 versus 0.49 ± 0.052; *P* = 5.1 × 10^−4^; pre-odd versus pre-even, 0.93 ± 0.011 versus −0.048 ± 0.048; *P* = 5.9 × 10^−6^). Paired two-sample two-sided *t*-test for both comparisons. Data are presented as mean ± s.e.m. Source data

Next, we sought to examine how experience influences the balance of space- and goal-referenced spatial representation in CA1 (ref. ^11^). We trained a separate cohort of mice with a fixed reward at 50 cm (*n* = 6 mice) on one belt (belt A, familiar), then exposed them to another belt covered with a set of unfamiliar tactile cues (belt B, novel). On the first day of exposure to the novel belt, mice quickly adapted their behavior (Fig. 2a). The PC representation of the novel belt also contained an over-representation around the reward site, similar to that of a familiar belt (Extended Data Fig. 1b,c). PCs on a novel belt, however, had somewhat less spatial information and reduced trial-by-trial reliability (Extended Data Fig. 1d,e), consistent with previous reports^4,47,58,59^.

To understand how the manipulation of the space-related reference frame (familiar to novel external cues) influences the balance of space- and goal-referenced PCs in CA1, we next switched the reward location on the second belt as previously described. Mice adapted their behavior as before (Fig. 2a), but in stark contrast to the balanced proportions of space- and goal-referenced PCs on a familiar belt (Fig. 2e), the fraction of goal-referenced PCs on a novel belt was nearly ten-times that of space-referenced PCs (Fig. 2b–d and Extended Data Fig. 1g,i,k). Thus, PF displacement on a novel belt was heavily skewed toward 90 cm (Fig. 2e), and notably, goal-referenced PCs tiled the entire novel belt (Fig. 2b,c,f). Additionally, similar levels of intermediate PCs were found between familiar and novel environments (Extended Data Fig. 2c,d,f,g). Moreover, population spatial coding was reliable on a novel belt before the reward switch and exhibited goal-referenced dominance at all regions (Fig. 2g–i and Extended Data Fig. 1l,m). Thus, under our experimental conditions, the PC representation in CA1 is primarily goal-referenced in a novel environment.

**Extended Data Fig. 4:**
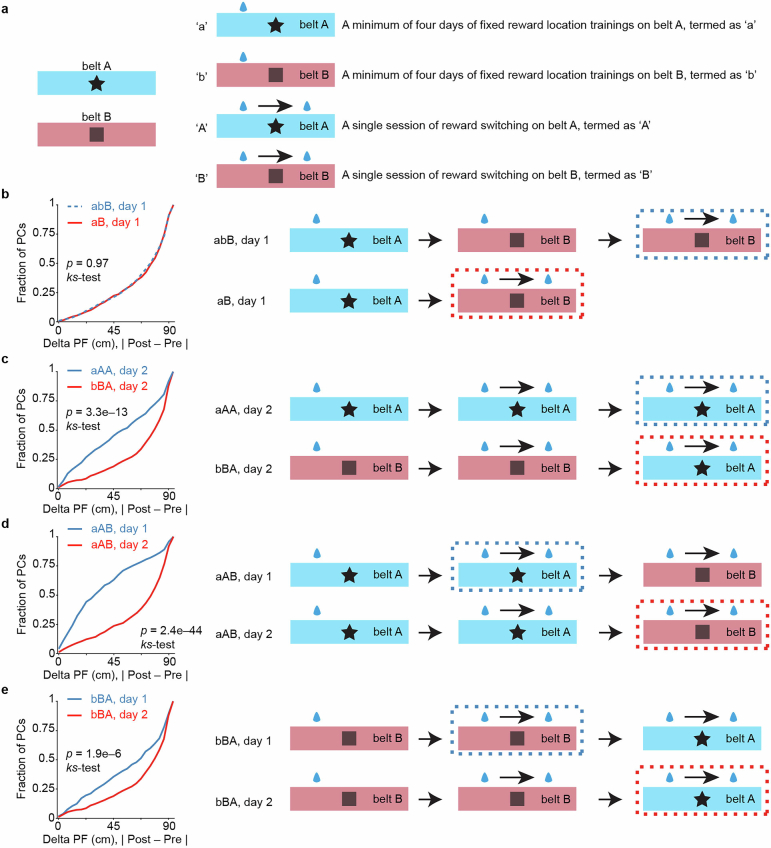
Experience-dependent, flexible space- and goal-referenced CA1 PC representation. **a**, Schematic of the naming conventions for training experiences and reward switch tests. Two environments, belt A and belt B, are utilized. Lowercase ‘a’ and ‘b’ represent ≥4 days of fixed reward training on belt A or B, respectively. Uppercase ‘A’ and ‘B’ represent a single reward switching session on belt A or B, respectively. For instance, ‘abB’ indicates fixed reward training on belt A for ≥4 days (‘a’), an additional 4-day fixed reward training on belt B (‘b’), and a reward switch test on belt B (‘B’, Familiar, Day 1). ‘aB’ represents fixed reward training on belt A (‘a’) followed by a reward switch test on belt B (‘B’, Novel, Day 1). **b**, Cumulative PC fractions across PF position changes between Post and Pre for ‘aB’ (red, 425 PCs from 6 mice) versus ‘abB’ (blue, 849 PCs from 4 mice) on Day 1. *p* = 0.97. For ‘aB’ and ‘abB’, Day 1 is the first day on belt B with a reward switch. For ‘abB’, two out of four mice had prior reward switching experiences. **c**, Same as **a** but for groups ‘aAA’ on Day 2 (blue, 368 PCs from 6 mice) versus ‘bBA’ on Day 2 (red, 375 PCs from 3 mice). *p* = 3.3e–13. **d**, Same as **a** but for groups ‘aAB’ on Day 1 (blue, 503 PCs from 5 mice) versus Day 2 (red, 489 PCs from 5 mice). Group ‘aAB’ was trained on belt A (‘a’), followed by a reward switch on belt A (Day 1, Familiar ‘A’) and belt B (Day 2, Novel ‘B’). *p* = 2.4e–44. **e**, Same as **d** but for groups ‘bBA’ on Day 1 (blue, 403 PCs from 3 mice) versus Day 2 (red, 375 PCs from 3 mice). Group ‘bBA’ was initially trained on belt B, followed by a reward switch on belt B (Day 1, Familiar ‘B’) and belt A (Day 2, Novel ‘A’). *p* = 1.9e–6. Note: Two-sample two-sided Kolmogorov–Smirnov test for panels **b**–**e**. Source data

Of note, an additional four consecutive days of training on the belt B did not result in balanced space- and goal-related referencing (*n* = 4 mice; Extended Data Fig. 4a,b). The number of training laps on the belt B closely matched that of the familiar belt A (763.3 ± 21.2 versus 700.0 ± 40.5 laps; *P* = 0.27, unpaired two-sided *t*-test), suggesting that the decreased proportion of space-referenced PCs on the second belt was not due to the novelty of the belt per se nor a lack of experience. Also, the distinct space- and goal-referenced PC proportions on the familiar and novel belts were not due to the animals’ inherent preferences for specific tactile cues associated with each belt^60^ (*n* = 3 mice; Extended Data Fig. 4a,c). By counterbalancing the presentation order of belts and conducting reward-switch experiments on two consecutive days (familiar belt A to novel belt B versus familiar belt B to novel belt A), we observed a consistent trend that PCs in the familiar condition remained significantly more space-referenced than the novel condition, independent of the order in which the belts were presented (Extended Data Fig. 4a,d,e). Together, these results demonstrate that experience alters the balance of goal- and space-related referencing present in the spatial representation of environments in CA1, and that exposure to a second environment reshapes the representation to become primarily goal-referenced.

### Individual PCs change referencing

**Fig. 3:**
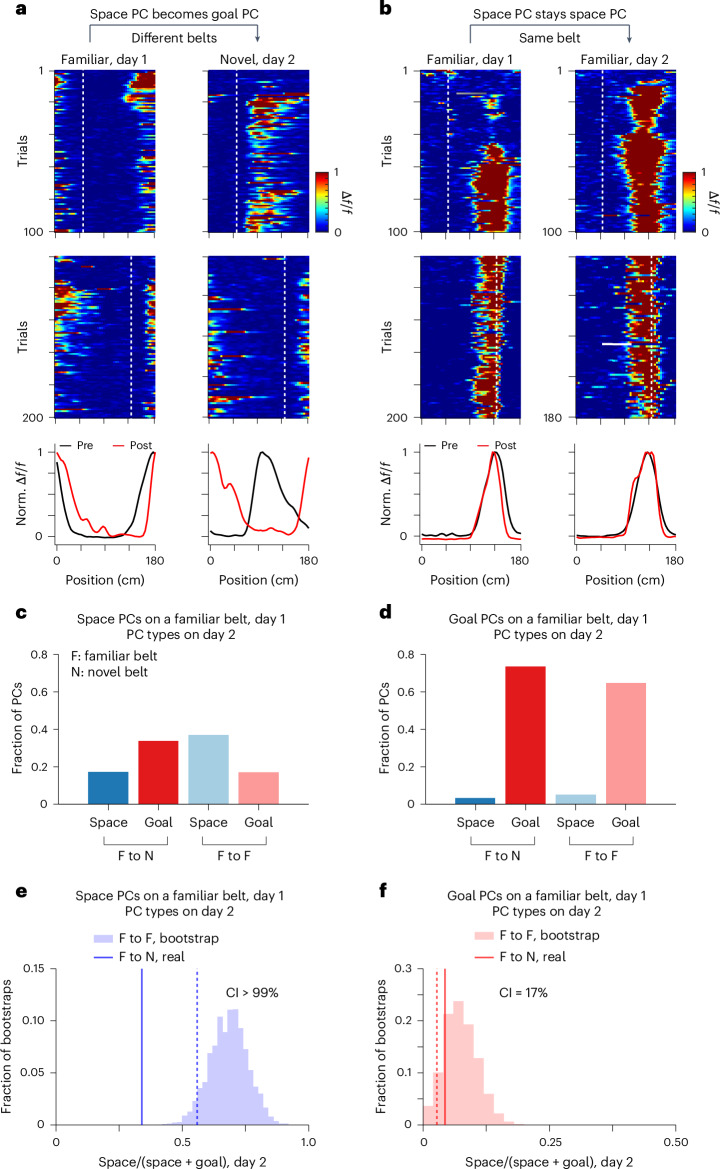
Individual PCs changed reference frames. **a**, An example of a space PC becoming goal-referenced. Heatmaps depict the spatial Δ*f*/*f* activity across laps for day 1 on a familiar belt (left) and day 2 on a novel belt (right) during pre (top) and post (middle). White dashed lines indicate reward locations (pre, 50 cm; post, 140 cm). Peak-normalized spatial Δ*f*/*f* during pre and post on day 1 (familiar, left) and day 2 (novel, right) (bottom). The heatmaps use smaller color scales (0–1) to visualize relatively low activity from individual cells. **b**, Same as **a**, but showing a PC that remained space-referenced on the same familiar belt for two consecutive days. **c**, Stability of space PCs across 2 days. The fraction of space PCs (day 1, familiar belt) that stayed space-referenced (dark blue) or switched to goal-referenced (dark red) on day 2 on a novel belt (left). The fraction of space PCs (day 1, familiar belt) that stayed space-referenced (light blue) or became goal-referenced (light red) on day 2 on the same familiar belt (right). **d**, Same as **c**, but showing the stability of goal PCs across 2 days. **e**, Resampled bootstrapped values of the ‘space/(space + goal)’ index across 2 days (F to F, familiar to familiar). The ‘space/(space + goal)’ index is calculated as the number of space PCs that stayed space-referenced divided by the total number of space PCs that stayed space-referenced or became goal-referenced on the next day. For each bootstrap, we randomly sampled a fraction of the real data with replacement to calculate the space/(space + goal) index using PCs on day 1. The dashed line represents the bottom fifth percentile of the bootstrapped values during F to F. The solid line represents the observed proportion from F to N (familiar to novel). **f**, Same as **e**, but showing the stability of goal PCs across 2 days. *n* = 6 mice for all conditions shown in **c** and **d**. Source data

The above data suggest that individual PCs may change their referencing. To examine this we determined, as above, the PC referencing over multiple days under the two environmental conditions (day 1/day 2; familiar/familiar and familiar/novel). On the first day, we trained a set of mice on a familiar belt, switched the reward on this belt and categorized the referencing of the recorded PCs (familiar, day 1; Fig. 3a,b). On the next day (day 2), these mice were split into two groups: one that experienced a reward switch on the same familiar belt (familiar/familiar) and the other that experienced the reward switch on a novel belt (familiar/novel). We recorded the same PCs on each belt and determined their referencing (day 2; Fig. 3a,b).

**Extended Data Fig. 5:**
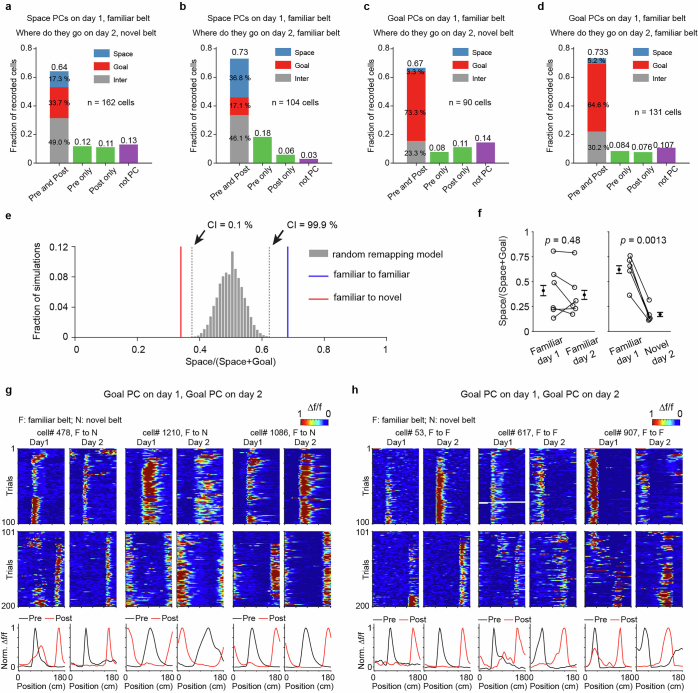
Longitudinal tracking of cells from day 1 to day 2. **a**, The fraction of Space PCs from a familiar belt (Day 1) classified as different cell types on Day 2 on a novel belt (162 Space PCs, 5 mice). **b**, Same as **a**, but on the same familiar belt for two days (104 Space PCs, 6 mice). **c**, Same as **a**, but for Goal PCs from a familiar belt (Day 1) to a novel belt (Day 2). (90 Goal PCs, 5 mice). **d**, Same as **c**, but on the same familiar belt for two days (131 Goal PCs, 6 mice). **e**, Distribution of simulated Space/(Space+Goal) index using a random remapping model. ‘Space/(Space+Goal)’ index: Number of Space PCs that remained space-referenced divided by the total number of Space PCs that either remained space-referenced or became goal-referenced on the next day. n = 10,000 shuffles. Black vertical dashed lines indicate the 0.1^st^ (0.38) and 99.9^th^ (0.62) percentile of the simulations, respectively. Blue and red lines indicate the observed index values for ‘familiar to familiar’ (0.68) versus ‘familiar to novel’ conditions (0.34), respectively. CI: Confidence interval. n = 6 mice. **f**, Space/(Space+Goal) index (the fraction of Space PCs over the sum of Space and Goal PCs on each day) across days for familiar versus familiar (left, 0.41 ± 0.11 versus 0.37 ± 0.092; *p* = 0.48) and familiar versus novel belt conditions (right, 0.62 ± 0.069 versus 0.17 ± 0.038, *p* = 0.0013). Each dot represents one animal. n = 6 mice for all conditions. Paired two-sided student *t*-test for both comparisons. Data are presented in mean ± SEM. **g**, Three examples of stable Goal PCs for two days from a familiar belt (Day 1) to a novel belt (Day 2). Colormaps of spatial Δf/f in Pre (top) and Post (middle) for Day 1 (left) and Day 2 (right). Bottom: Peak-normalized (norm.) mean Δf/f in space for Pre and Post. Day 1 and Day 2: The first and second day with a reward switch, respectively. **h**, Same as **g**, but on the same familiar belt. Source data

Of the space-referenced PCs on a familiar belt that remained PCs both before and after the reward switch on a novel belt (familiar/novel; 64% of all space-referenced PCs; Extended Data Fig. 5a), 34% became goal-referenced (Fig. 3a), twice as many as those that remained space-referenced (17% stayed space-referenced; Fig. 3c). In contrast, when the same familiar belt was employed on both days, 37% of space-referenced PCs that remained PCs on the same familiar belt on the next day remained space-referenced (familiar/familiar; 73% of all space-referenced PCs; Fig. 3b and Extended Data Fig. 5b), whereas 17% became goal-referenced (Fig. 3c). Therefore, space-referenced PCs remained relatively stable on the same belt across 2 days, with a small but noticeable proportion of reference frame switching (Fig. 3e and Extended Data Fig. 5e,f); however, the introduction of a second belt markedly increased the rate of reference frame switching from space-referenced PCs to goal-referenced PCs (Fig. 3c,e; CI > 99%; Extended Data Fig. 5e,f). On the other hand, goal-referenced PCs on day 1 remained stable on the next day for both conditions (Fig. 3d,f and Extended Data Fig. 5c,d,g,h). These results demonstrate that experience flexibly shapes individual PCs to switch their references from space- to goal-related reference frames.

### PCs receive both goal- and space-referenced synaptic inputs

**Fig. 4:**
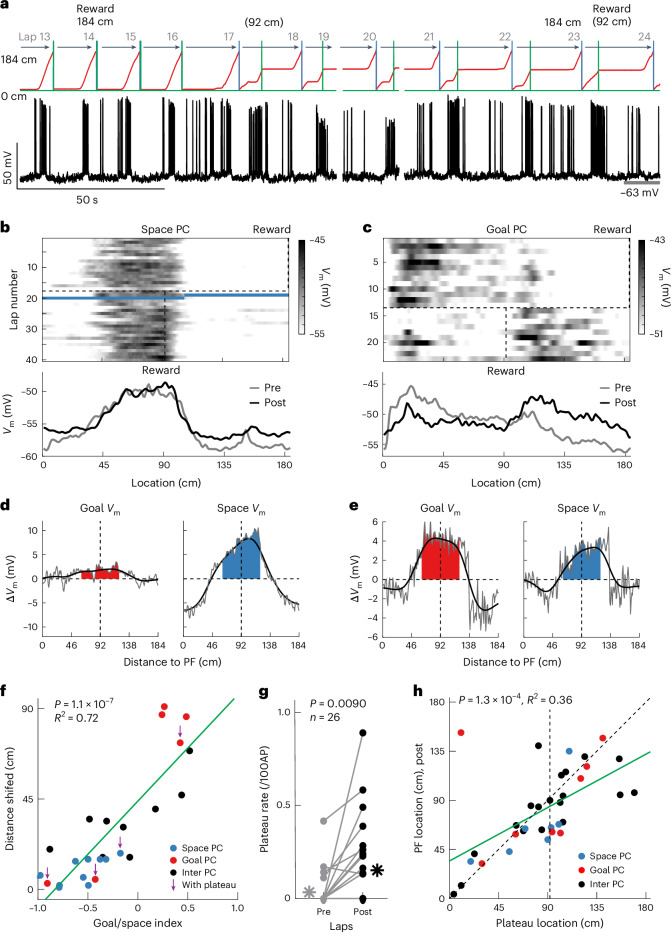
Individual PCs receive both goal- and space-referenced synaptic inputs. **a**, An example *V*_m_ trace of a space PC (black) and mouse locations (red, from 0 to 184 cm). Green lines mark reward locations (pre, 184 cm; post, 92 cm). Blue lines indicate lap reset locations. Pre and post indicate before and after a reward switch, respectively. **b**, A representative space PC during the reward switch (same cell as in **a**). The shaded heatmap shows the *V*_m_ in space across laps (top). Dashed vertical lines indicate reward locations (pre, 184 cm; post, 92 cm). The dashed horizontal line marks the trial of reward switch. Laps 19 and 20 have blue-colored blanks due to access changes, which are also excluded in **a**. Averaged *V*_m_ traces in pre (gray) and post (black) (bottom). **c**, Same as **b**, but for a representative goal PC. **d**, Δ*V*_m_ produced by the reward switch for *V*_m_ traces referenced to the space (red, goal *V*_m_) or to the running start (blue, space *V*_m_), corresponding to the space PC in **b**. **e**, Same as **d**, but for the goal and space *V*_m_ corresponding to the goal PC in **c**. **f**, The correlation between goal/space index and the PF shift distance after the reward switch but before spontaneous plateaus. Purple arrows indicate cells that changed their categories after spontaneous plateaus. The black, blue and red dots indicate the intermediate, space- and goal-referenced PCs, respectively. The green line indicates a two-sided linear fit (*n* = 25 cells, *P* = 1.1 × 10^–7^, *R*^2^ = 0.72). **g**, The plateau rate per 100 spikes in pre (gray) and post (black). The far left and right asterisks represent the averages (*n* = 26 cells, *P* = 0.0090, paired-sample two-sided *t*-test). **h**, The correlation between spontaneous plateau locations and PF positions after the given plateaus. The black, blue and red dots indicate the intermediate, space- and goal-referenced cells, respectively. The green line is a two-sided linear fit (*n* = 35 identified plateaus, *P* = 1.3 × 10^–4^, *R*^2^ = 0.36). Source data

**Extended Data Fig. 6:**
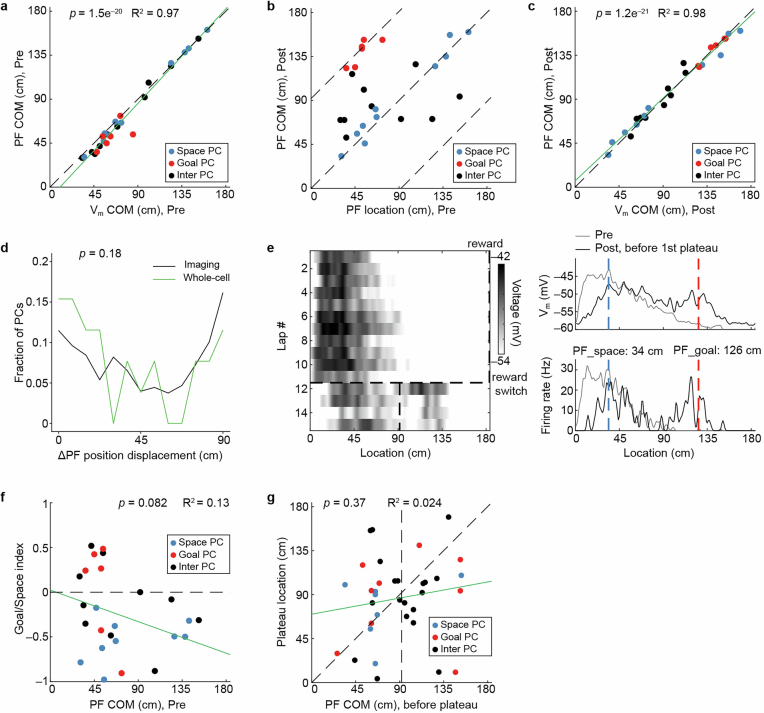
Additional characteristics of CA1 PC remapping. **a**, The correlation between the V_m_ COM and PF COM in Pre. The green line is a two-sided linear fit (n = 26 cells, *p* = 1.5 × 10^–20^, R^2^ = 0.97). **b**, PF remapping of 26 recorded CA1 PCs. The reward is at 184 cm and 92 cm before (Pre) and after (Post) the reward switch, respectively. The dashed lines indicate the distribution center of goal- and space-referenced cells. The black, blue, and red dots indicate the intermediate, space-, and goal-referenced PCs, respectively. **c**, The correlation between the V_m_ COM and PF COM in Post. The green line is a two-sided linear fit. (n = 26 cells, *p* = 1.2 × 10^–21^, R^2^ = 0.98). **d**, The fraction of PCs as a function of shifted PF distance after the reward switch. The black and green lines represent imaging and whole-cell data, respectively (*p* = 0.18, two-sample two-sided Kolmogorov–Smirnov test). **e**, An example cell demonstrating the split of a place field into goal- and space-referenced V_m_ ramps immediately following reward switch. Left: The shaded heatmap shows the V_m_ in space across trials before (Pre) and after (Post) the reward switch. The dashed lines indicate the reward locations (at 184 and 92 cm in Pre and Post, respectively). Right: The averaged V_m_ of Pre (gray) and Post (black) trials. The blue and red dashed lines indicate the original (space-referenced) PF and the Goal PF (92 cm away from the original PF), respectively. Bottom: The mean spatial firing rate of the cell in Pre (gray) and Post (black) trials. **f**, The Goal/Space index as a function of PF COM in Pre. The reward is at 184 cm in Pre. The green line is a two-sided linear fit (n = 25 cells, *p* = 0.08, R^2^ = 0.13). **g**, The correlation between the PF COM before spontaneous plateaus and the location of spontaneous plateaus. The green line is a two-sided linear fit (n = 35 plateaus, *p* = 0.37, R^2^ = 0.024). Source data

We next questioned what the mechanisms controlling PC referencing are. Given that modifications in the strength of CA3 synaptic input produce the membrane potential (*V*_m_) depolarizations that drive CA1 PF activity^61–64^, we performed intracellular whole-cell *V*_m_ recordings^62,65^ from CA1 pyramidal neurons of mice running on a familiar belt to examine how the synaptic input to PCs was affected by reward location changes (Fig. 4a). In all recorded neurons intracellular currents were injected to induce new PFs as previously reported^61,62^. Consistent with previous intracellular recordings^62,65–67^, these PCs exhibited slow ramps of depolarization that drove PF firing at selective locations (*V*_m_ ramps; mean ramp amplitude, 8.89 ± 0.64 mV, *n* = 26 cells; Extended Data Fig. 6a). We next switched the reward site, as above, and found that these PCs showed diverse PF remapping (6 of 26 were goal-referenced, 10 of 26 were space-referenced and 10 of 26 were intermediate; Extended Data Fig. 6b). After the reward switch, the location of *V*_m_ ramps was strongly correlated with the location of PF firings (Extended Data Fig. 6c), and the distribution of PF displacement was consistent with that of PCs recorded via two-photon Ca^2+^ imaging (Extended Data Fig. 6d).

**Extended Data Fig. 7:**
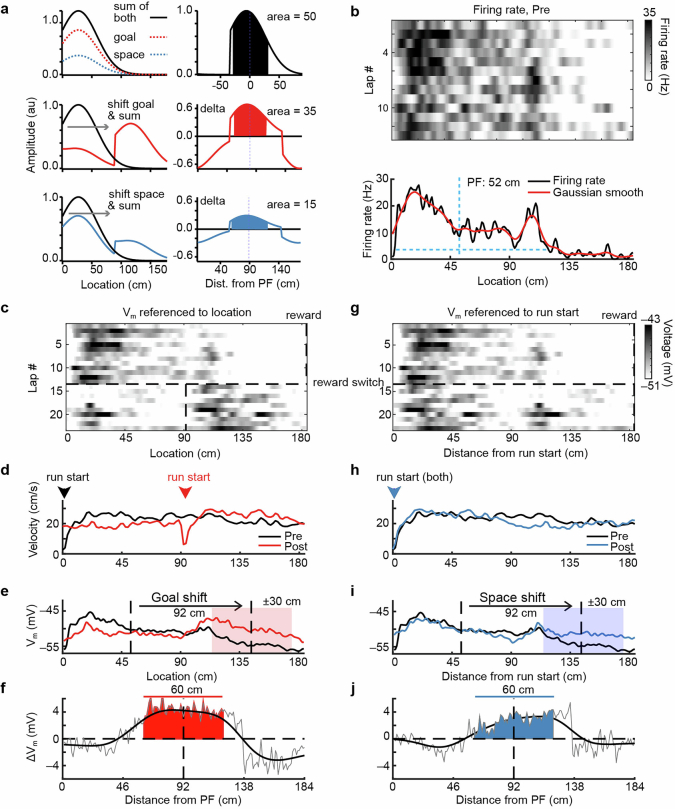
Calculating the putative goal- and space-referenced V_m_ by subtraction. **a**, Illustration of Goal and Space V_m_ calculation. Top: A PF V_m_ (black, area=50) comprising two components: Goal (red) and Space V_m_ (blue). Middle: Summation of the Space V_m_ and shifted Goal V_m_ (left red) allows the Goal V_m_ to be calculated from the subtraction of the left two traces (right red, shifted V_m_, area=35, aligned to original PF). Bottom: Summation of the Goal V_m_ and shifted Space V_m_ (left blue) reveals the Space V_m_ by subtracting the left two traces (right blue, shifted V_m_, area = 15, aligned to original PF). **b**, Top: Example PC firing rate heatmap in Pre. Bottom: Averaged firing rate (black) with Gaussian smoothing (red). Blue horizontal and vertical lines: Width and COM of PF. **c**, A spatial V_m_ heatmap (same cell in b) referenced to physical locations in Pre and Post. Dashed lines: Reward locations (184/92 cm) and the reward switching lap (Laps 13–14). **d**, Averaged running trajectories in Pre (black) and Post (red). Note that the running start shifted ~92 cm after the reward switch. **e**, Averaged V_m_ traces from c (Pre: black; Post: red). Dashed lines indicate that the COM of Goal V_m_ (144 cm) shifted 92 cm from the original PF (52 cm) because the running start shifted in Post in d. Red shading (±30 cm around the COM of the shifted Goal V_m_) marks the Goal V_m_ area. **f**, Subtraction of Pre and Post V_m_ traces from e, aligned to the PF COM in Pre (0 cm). Gray and black lines: raw and gaussian-smoothed traces. The red shading area quantifies the Goal V_m_ magnitude. **g**–**j**, Similar to **c**–**f**, but V_m_ traces are referenced to the running start. Now the reward and running start location remain at 184 and 0 cm during Pre and Post. Note that the V_m_ in Post in g shifted 92 cm compared to that in c. Blue shading area in j (±30 cm around the COM of shifted Space V_m_ subtracted from i) quantifies the Space V_m_ magnitude. Source data

**Extended Data Fig. 8:**
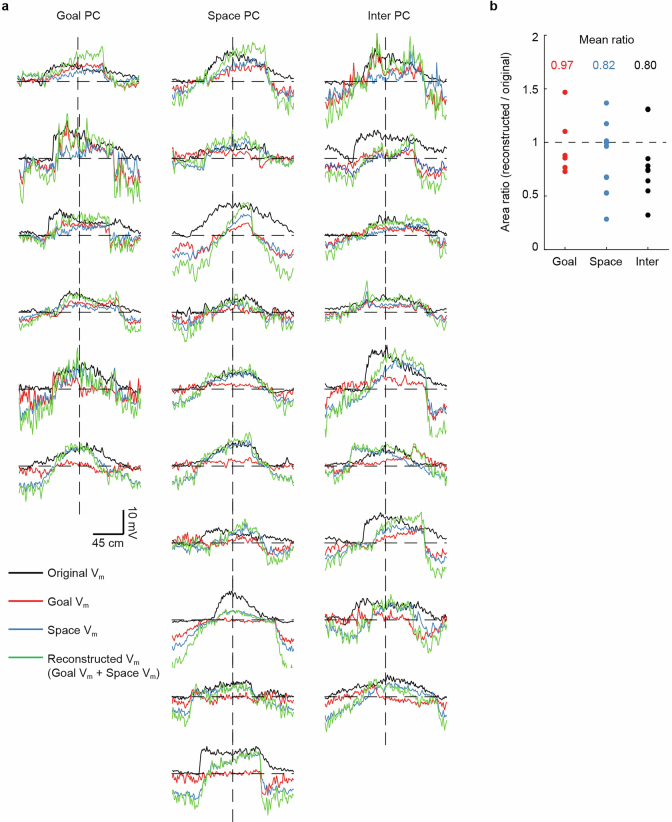
The reconstructed PF V_m_ and original PF V_m_ in individual CA1 PCs. **a**, The original, Goal, Space, and reconstructed V_m_ (Goal V_m_ + Space V_m_) in 25 recorded CA1 PCs grouped by the PC categories. Note that one cell is excluded due to an immediate spontaneous plateau after the reward switch (see Methods). **b**, The area ratio of the reconstructed PF V_m_ versus the original PF V_m_ in Goal, Space, and Inter PCs. The mean ratios are 0.97, 0.82, and 0.80 for Goal, Space, and Inter PCs, respectively. Source data

In general, we observed a ~90-cm shift of the *V*_m_ ramps of goal-referenced PCs (Fig. 4c), whereas the *V*_m_ ramps of space-referenced PCs remained at the original PF location (Fig. 4b). We did, however, also notice that in most neurons, the shape of the *V*_m_ ramp was altered after the reward switch as if the synaptic input underlying the PF was composed of separate components: one that shifted (goal-referenced input) and another that did not (space-referenced input) (Extended Data Fig. 6e). To quantify the ratio of these goal- and space-referenced inputs, we calculated a Δ*V*_m_ under two different referencing conditions (Fig. 4d,e and Extended Data Fig. 7). In the first condition the *V*_m_ was referenced to the physical locations of the track (0-cm belt location; space-referenced frame; Extended Data Fig. 7b–e), and in the second the *V*_m_ was referenced to the beginning of running on each lap following reward consumption (goal-referenced frame; Extended Data Fig. 7g–i). In the second condition, the *V*_m_ is essentially rotated 90 cm with respect to the first condition as the running profile is shaped by the reward location, which has itself been rotated 90 cm (Methods). In both referencing conditions, we subtracted the mean *V*_m_ traces calculated from all trials before the reward switch and after any spontaneous plateaus (Methods, ≥4 trials, 15.08 ± 0.63 trials) from the mean *V*_m_ calculated from all the trials after the reward switch and before any spontaneous plateaus (Methods, ≥3 trials, 19.88 ± 2.48 trials) for all PCs (Δ*V*_m_, *n* = 25 cells). The goal-referenced component of the *V*_m_ was determined as the amount of depolarization (area) that shifted 90 ± 30 cm away from the original PF location for the traces referenced to location (space-referenced frame; Extended Data Fig. 7a,e,f). The space-referenced component was determined as the amount of depolarization (area) that shifted 90 ± 30 cm for the traces referenced to the running start point (goal-referenced frame; Extended Data Fig. 7a,i–j). Summation of the two resulting components adequately reconstructed the original *V*_m_ in most cells, suggesting that this was a fairly accurate measure (mean deviation, 0.29 ± 0.037; Extended Data Fig. 8).

**Extended Data Fig. 9:**
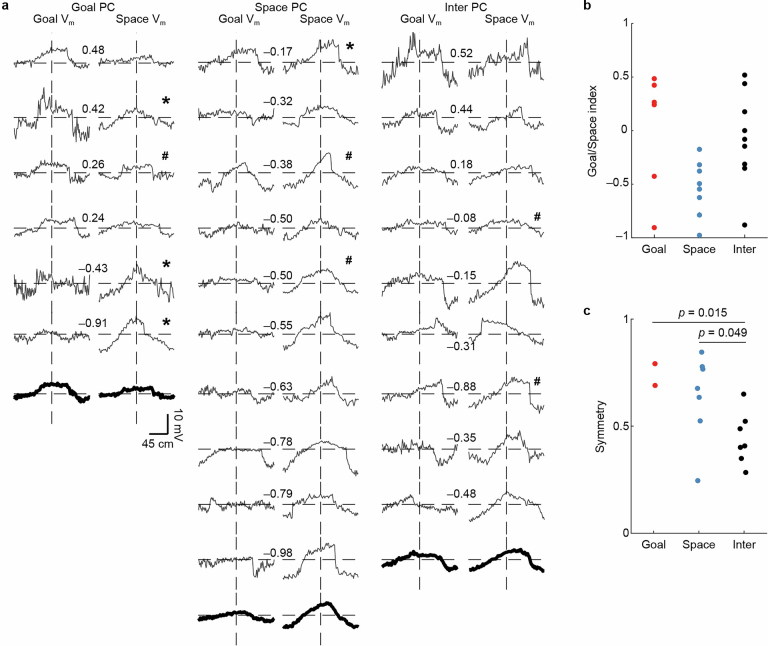
Putative goal- and space-referenced V_m_ in individual CA1 PCs. **a**, The Goal and Space V_m_ in 25 recorded CA1 PCs grouped by the PC categories. Note that one cell is excluded due to an immediate spontaneous plateau after the reward switch (see Methods). The number indicates the Goal/Space index of a given cell. The bold traces at the bottom are the averaged V_m_ for each individual column (cells marked by the star and # are excluded). The star marks the cells that changed their categories due to spontaneous plateau potentials. The # symbols mark the cells with two separate PFs (see Methods). **b**, The Goal/Space index in Goal, Space, and Intermediate cells. **c**, The symmetry of the total V_m_ (Goal V_m_ + Space V_m_) in Goal, Space, and Intermediate cells (the 9 cells marked by the star and # symbol in **a** are excluded. 0.74 ± 0.051, 0.64 ± 0.077 and 0.44 ± 0.046 for Goal, Space, and Intermediate PCs, respectively; *p* = 0.015 and 0.049 for Goal versus Inter and Space versus Inter PCs, respectively. Unpaired two-sided *t*-test for both comparisons). Source data

We calculated a goal (G)/space (S) index ((G – S)/(G + S)) from these quantities and found a strong correlation between the amount the PF shifted and the index, with goal-referenced PFs showing positive values, space-referenced PFs showing negative values and intermediate PF with values in between these extremes (Fig. 4f and Extended Data Fig. 9a,b). The location dependence of the goal/space index was relatively consistent with that observed in the imaging data (Extended Data Fig. 6f). Together, these results suggest that CA1 PCs simultaneously receive both goal- and space-referenced synaptic inputs and that the relative proportion of goal/space inputs is a determinant of the distance PFs initially shift. In addition, a level of imperfect stability or shifting may also contribute to the PF locations of some intermediate PCs (Extended Data Fig. 9c). Thus, individual PCs in CA1 receive a variable proportion of both goal- and space-referenced synaptic inputs, which largely dictate the initial remapping following a reward switch.

### BTSP also determines some place field locations

**Fig. 5:**
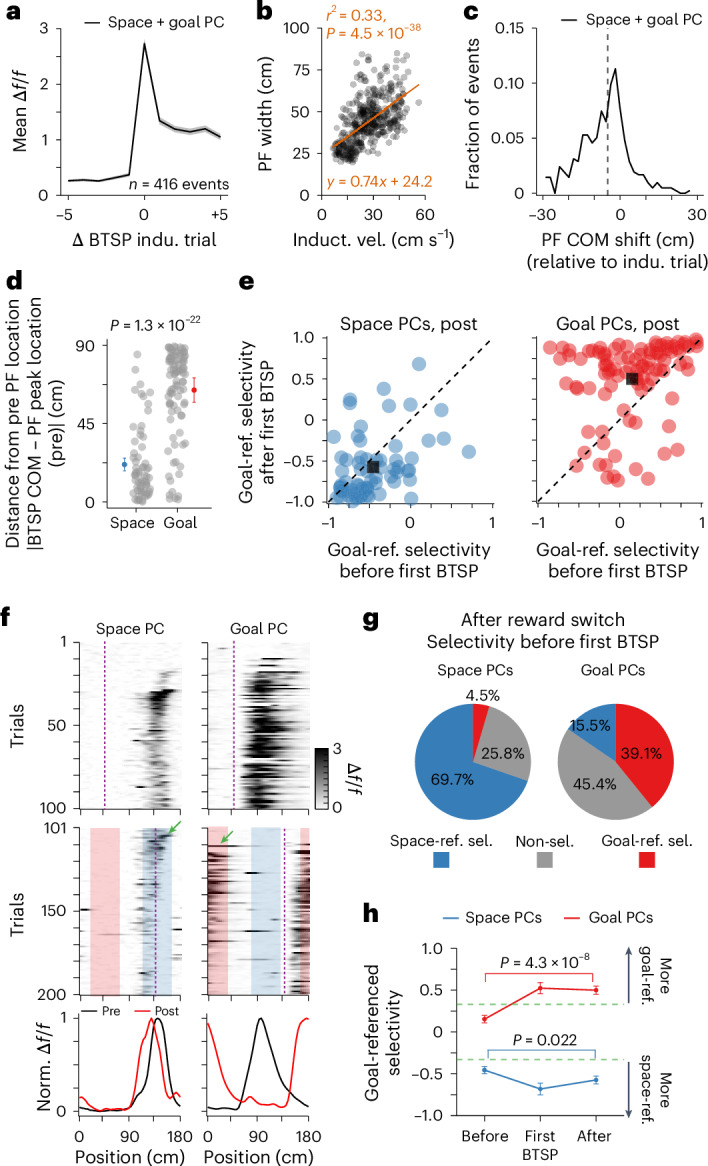
BTSP was involved in PC remapping. **a**, Mean in-field Δ*f*/*f* across trials aligned to the BTSP induction (indu.) trial during pre (*n* = 416 BTSP events, six mice). Shading shows s.e.m. **b**, PF width following BTSP versus running velocity during BTSP induction. Each dot depicts one BTSP event. Induct. vel., induction velocity. Orange line shows the two-sided linear fit. **c**, Distribution of PF center of mass (COM) shifts after the BTSP event. Black dashed line shows the median shift (−4.16 cm). Data included only goal- and space-referenced PCs (235 PCs) for **a**–**c**. **d**, Distance of the first BTSP event in post from the PF in pre for space (left) and goal (right) PCs. Each dot shows one BTSP event (space versus goal, 21.5 ± 2.6 cm versus 64.3 ± 2.5 cm; *P* = 1.3 × 10^−22^, unpaired two-sided *t*-test). **e**, Goal-referenced (ref.) selectivity before and after the first BTSP event during post for space-referenced (left, blue) and goal-referenced (right, red) PCs. Black square shows mean selectivity. Dashed line shows unity. Goal-referenced selectivity: Δ*f*/*f* difference between the goal-referenced and space-referenced field divided by their sum. Each dot depicts one BTSP event. **f**, Examples of space (left) and goal (right) PCs with BTSP events. Heatmaps depict spatial Δ*f*/*f* during pre (top) and post (middle). Green arrows show first BTSP events (post). Dashed lines show reward locations. Shaded areas (~45 cm wide) show space-referenced (blue, around the original PF in pre) and goal-referenced (red, 90 cm away from the original PF in pre) fields. Peak-normalized mean Δ*f*/*f* (bottom). **g**, Fractions of activity types before the first BTSP event in post for space-referenced (left) and goal-referenced (right) PCs (*n* = 6 mice). Criterion, >0.33, goal-referenced; <−0.33, space-referenced; in between, non-selective (non-sel.). Sel., selectivity. **h**, Goal-referenced selectivity before, during and after the first BTSP event for all space-referenced (blue) and goal-referenced (red) PCs in post. Space PC, before versus after, −0.46 ± 0.044 versus −0.57 ± 0.046, *P* = 0.022; goal PC, before versus after, 0.15 ± 0.045 versus 0.50 ± 0.049; *P* = 4.3 × 10^−8^. Paired two-sided *t*-test for both comparisons). Green lines show thresholds. All data are from the familiar belt, day 1 (six mice). Data show mean ± s.e.m. For **d**–**e** and **g**–**h**, *n* = 66 and 97 events for space and goal PCs, respectively. Source data

**Extended Data Fig. 10:**
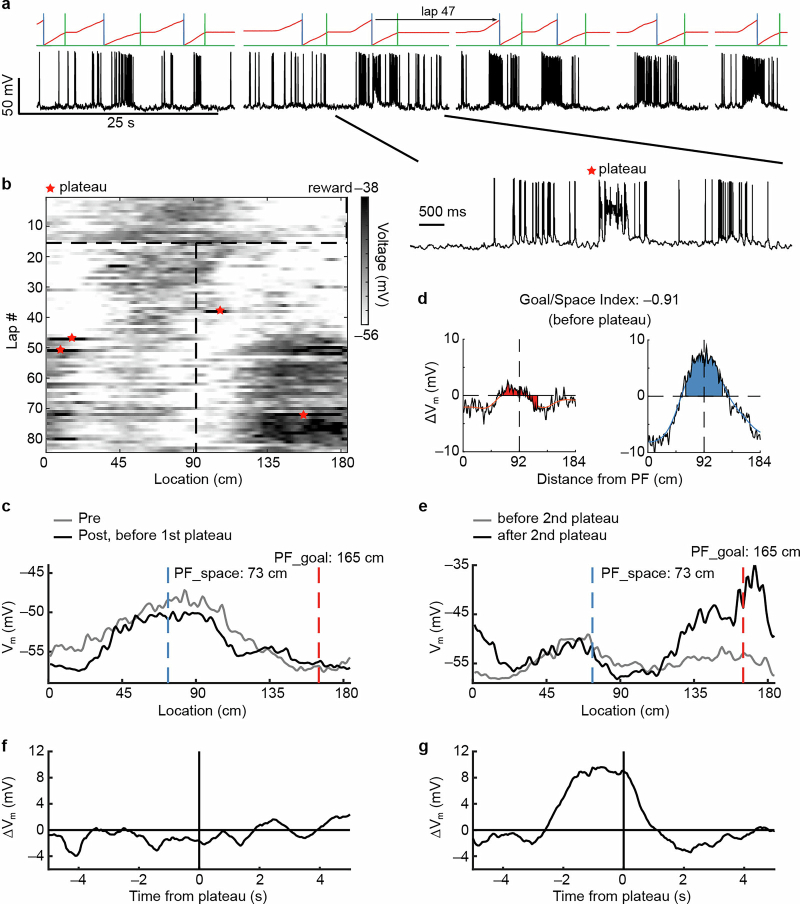
Spontaneous plateau potential initiation after the reward switch. **a**, V_m_ of a PC with spontaneous plateau potentials (black). The red line indicates the location of the mouse on the belt (from 0 to 184 cm). The green line indicates the reward location (92 cm in Post), and the blue lines indicate the end of the lap (184 cm). Bottom: The expanded V_m_ trace showing the spontaneous plateau potential at the beginning of lap 47. **b**, A shaded heatmap shows the V_m_ across trials in Pre and Post. The vertical dashed lines indicate the reward locations (at 184 and 92 cm in Pre and Post, respectively). The horizontal dashed line marks the trial of the reward switch. The red stars indicate the spontaneous plateau potentials. **c**, The averaged V_m_ traces of Pre (gray, lap 1–15) and Post (black, before the first plateau, lap 16–37) trials. The blue and red dashed lines indicate the original PF (space-referenced) and the Goal PF (92 cm away from original PF). **d**, The putatively goal- (left, red) and space-referenced (right, blue) V_m_ of this example cell. **e**, The averaged V_m_ traces before (gray, trials 39–46) and after (black, lap 48–50) the 2nd plateau potentials (at lap 47). The blue and red dashed lines indicate the original PF (space-referenced) and the Goal PF (92 cm away from original PF). **f**–**g**, The BTSP kernels of the first (**f**, lap 38) and second (**g**, lap 47) plateau potentials. Source data

In our *V*_m_ recordings, approximately 46% of PCs (12 of 26) exhibited spontaneous long-duration plateau potentials^62,68^ that altered the location of the PFs by rapidly overwriting the current synaptic weights^69^ (Fig. 4h and Extended Data Fig. 6g and examples in Extended Data Fig. 10). Indeed, the plateau potential rate significantly increased after the reward switch (from 0.003 to 0.15 per 100 spikes; *P* = 0.0090, paired *t*-test; Fig. 4g), suggesting that an enhanced level of behavioral timescale synaptic plasticity (BTSP) could also play a role in PC remapping^70^. To further explore how BTSP modulates PC remapping, we identified putative BTSP events from the above population imaging data recorded under familiar conditions (Fig. 5a–c and Methods). With our criteria, we found BTSP events after the reward location switch in 63.5% of space-referenced PCs and 74.1% of goal-referenced PCs. In most space-referenced PCs with BTSP events (69.7%), the events were clustered around the original PF location where the apparent plasticity induction enhanced the space-referenced selectivity (Fig. 5d–h). In the remaining space-referenced PCs with BTSP events (30.3%), the plasticity was primarily responsible for producing the space-related referencing, as the activity of these neurons had become non-specific or goal-referenced initially after the reward switch (Fig. 5g). For goal-referenced PCs with BTSP events, the events were preferentially clustered nearly 90 cm away from the original PF location (Fig. 5d). In approximately three-fifths of goal-referenced PCs with BTSP events (60.9%), BTSP in a single trial shifted the PF location to produce goal-referenced activity while it enhanced the level of goal-referenced selectivity already present in the remaining 39.1% of goal-referenced PCs (Fig. 5e–h). Together, the above data suggest that goal- and space-referenced synaptic inputs support a conjunctive map in CA1, with appropriate shifting of these synaptic inputs and subsequent BTSP induction both contributing to the remapping observed following the reward location switch.

## Discussion

### Summary

Here we report that both goal- and space-related reference frames contribute to a conjunctive spatial map of a familiar environment. We find this conjunction is apparent at both the population level (goal- and space-referenced PCs) and the cellular level (goal- and space-referenced synaptic inputs to PCs). We also report that experience can reshape the conjunctive map, as exposure to a second environment leads to individual PCs flexibly changing reference frames mainly from space- to goal-referenced. Moreover, the dynamic PC remapping in CA1 after the reward switch seems to be mediated by a combination of factors such as alterations in the location dependence of synaptic input (perhaps explained by changes in the activity of area CA3) and new synaptic plasticity in CA1 rapidly produced by dendritic plateau potentials (BTSP).

### A conjunctive spatial map in CA1

We find that, when an animal is engaged in a goal-directed spatial learning task in a familiar environment, a conjunctive cognitive map in hippocampal area CA1 is instantiated by a mixture of goal- and space-referenced PCs. Therefore, our findings favor the hypothesis that multiple reference frames support a spatial map in the hippocampus^2,34,35,38^.

In our task, goal-referenced PCs tend to cluster after, but not before, the reward site. For several reasons, it is unlikely that these goal-referenced PCs represent direct responses to the reward. First, the majority of goal-referenced PCs have their PFs located more than 30 cm in space following the reward site. Second, following the reward delivery there is an approximately 6-s stationary period before an animal starts to run for the next lap, and this period was excluded from our PC analysis. A recent study using a similar task found a reward consumption signal in LEC that was more transient (<3 s) than our stationary period, and furthermore this signal was not prominent in CA1 (ref. ^42^). Third, goal-referenced PCs that cluster after the reward site reliably encode spatial, but not temporal, information after running onset; thus, our results do not favor the hypothesis that they represent a strictly time-dependent process after running. How these goal-referenced PCs are formed from the beginning of experiences remains an exciting question for future studies.

The reward site may, however, function as a starting point to update an animal’s present position primarily through the process of path integration^12,21,23,53^. Together, these goal-referenced PCs resemble those PCs that cluster at the beginning of an animal’s path^32,34,56^, potentially providing a distance metric away from the previous goal location. As the animal moves further away from its starting position, we find that the PC representation is composed more heavily of PCs referenced to spatial cues which can, in principle, correct the path integration drift error^21,23,53^. Notably, the fraction of space-referenced PCs has a strong inverse correlation with that of goal-referenced PCs, suggesting a competitive relationship^34^. Thus, goal- and space-referenced PCs together form a conjunctive map that supports different phases in our goal-directed spatial learning task.

### Experience-dependent flexible referencing

After mice have learned the spatial relationship between the reward and the spatial cues in a familiar environment, performing the same task in a second environment with unfamiliar external cues causes the PC representation to become predominantly goal-referenced. The PC remapping in the second environment after the reward switch can be explained by a shift in the goal-related reference frames. Moreover, our longitudinal recordings confirm that a large fraction of space-referenced PCs adaptively become goal-referenced PCs in this process. This is unlikely due to the novelty of the second environment per se, because further extensive training does not recover the balanced ratio of space- and goal-referenced PCs in CA1 in the second environment. Additionally, our results are consistent with previous work suggesting that path integration using self-motion cues alone is sufficient to maintain an established map^6,14,71^. Overall, our observations clearly demonstrate that the anchoring of individual PCs to either space- or goal-related reference frames is not fixed but is instead flexibly shaped by previous experience.

The underlying factors shaping the exact ratio of space- and goal-referenced PCs in our goal-directed task remain to be addressed; however, evidence from randomly foraging rats has shown that when the visual cues are perceived as unstable, CA1 PCs rely more on self-motion cues, suggesting that the mechanism governing PC referencing in CA1 is not a static property of the hippocampus but is instead shaped by previous experiences^44–46^. This adaptability might explain why, after experiencing more than one environment with the same task structure, animals learn to rely more on reward cues than spatial cues in subsequent environments during navigation between reward goals. Of note, the discovery of an experience dependence in PC referencing in these two distinct types of behavioral paradigms suggests a more general role of experience in integrating distinctly referenced components of the cognitive map in the hippocampus.

Hippocampal remapping has been proposed to reflect animals’ inferences of hidden environmental states^72^. Within this framework, the hippocampus forms a cognitive map through experience allowing animals to develop ‘subjective beliefs’ about their location in the environment by integrating available sensory information. This adaptive PC referencing suggests that, rather than simply encoding spatial locations in relation to immediate spatial cues, hippocampal PCs incorporate sensory inputs along with goals and memories of past experiences. This capability is particularly crucial in navigation through uncertain environments, where direct cues may be sparse, ambiguous or even absent^73^.

### Synaptic mechanisms

Previous studies revealed that dendritic plateau potential-driven BTSP adjusts the weights of synaptic inputs to produce the large *V*_m_ ramps that drive PF firing in CA1 (refs. ^61–63,69^). Our whole-cell recordings suggest that the majority of synaptic inputs with strong weights on CA1 PCs remain active after the reward switch, with these inputs either maintaining or immediately altering their location dependence. This indicates that new synaptic plasticity through BTSP is not necessarily required for the initial remapping process in many PCs. Rather, our data suggest that much of the immediate PF reorganization in CA1 after the reward switch is inherited from its upstream regions, presumably CA3 (refs. ^64,74,75^). Of note, *V*_m_ ramps of most PCs split into goal- and space-referenced components, indicating that individual CA1 PCs simultaneously receive both goal- and space-referenced synaptic inputs to produce their conjunctive PFs. The proportion of potentiated goal- and space-referenced synaptic inputs that each PC receives then primarily determines the degree of PF shifting in these PCs.

We also find, however, that the reward switch elevates the rate of plateau potential initiation in CA1, and that this rapidly reshapes the PF referencing of many PCs by overwriting the synaptic weight matrix through BTSP^69^. Further, our data suggest that BTSP might preferentially enhance the PFs of some space-referenced PCs and may also abruptly form a large fraction of the goal-referenced PC population. Together, we propose that the reorganization of space- and goal-referenced feedforward CA3 synaptic inputs and the induction of new BTSP collectively produce the PC remapping observed in CA1 after the reward switch.

## Conclusion

Understanding how different reference frames are encoded and retrieved in the hippocampus could ultimately help improve our mechanistic understanding of how a cognitive map is formed and retrieved there^1,2,10–12^. It is commonly believed that the hippocampus receives distinctly referenced synaptic inputs from its upstream neocortical regions MEC and LEC^41,42,76^. Our observation that CA1 PCs are formed by simultaneous input from both goal- and space-referenced synaptic activity suggests that these reference frames are integrated—perhaps for the first time—within CA1 to produce a conjunctive yet coherent spatial map. But this remains an open question, as future experiments are needed to determine PC referencing in upstream regions particularly in area CA3. Therefore, we suggest that PC remapping in CA1 can be, at least partially, explained by a dynamic reorganization of distinctly referenced synaptic inputs. Finally, that the degree of this reference frame mixing being dependent upon the experience of the animal suggests that mechanisms exist to link the experience of the animal with the individual components of the cognitive map^11,13,63^.

## Methods

All experimental procedures performed were approved by the Baylor College of Medicine Institutional Animal Care and Use Committee (protocol AN-7734).

### Surgery

All experiments were performed in adult GP5.17 (ref. ^77^) (*n* = 22 mice, ≥8 weeks old at surgery, of either sex) or wild-type male C57BL/6 mice (*n* = 16 mice, 8–12 weeks old). Animals were housed in the Magee Satellite under an inverse dark–light cycle (light, 21:00 to 9:00), with controlled temperature (~21 °C) and humidity (30–60%). All recordings were carried out during the dark cycle. All mice were anesthetized with ~2% isoflurane for surgeries which were performed as previously described^48,78^. After applying local anesthetics, a small flap of scalp skin was removed, and the skull cleaned and leveled. Craniotomy center was marked for CA1 imaging (in mm) at 2.3 (anterior–posterior, posterior to bregma) and 2.15 (medial–lateral, lateral to midline) or for intracellular recordings at 1.9 (anterior–posterior) and 2.0 (medial–lateral, right hemisphere). The surgery procedure for intracellular recordings followed previous detailed reports^61,62^. For two-photon imaging, a 3.0-mm-diameter craniotomy was made centered as described. Next, the dura was removed using forceps (Fine Science Tools) and the overlying cortical tissue was gently aspirated using a blunt needle (McMaster-Carr) under repeated irrigation. Aspiration exposed the external capsule, which was lightly peeled without direct needle contact. Saline irrigation continued until the bleeding ceased. A cannula (3-mm diameter, 1.75–2.0-mm long) with a cover glass (Potomac, 2.90-mm diameter, number 0) glued to the bottom using UV-curable optical adhesive (Norland Products) was inserted and cemented. The cannula was tightly fit to the craniotomy for better stability. Finally, we used dental cement (Ortho-Jet, Lang Dental) to secure a custom-made titanium head bar to the skull, parallel to the cover glass surface for optimal imaging quality.

### Behavior task (two-photon imaging)

The linear treadmill system consists of a ~180-cm velvet fabric belt (McMaster-Carr)^49,79^. Mice were head-fixed to custom-fabricated stainless-steel head posts. The belt was self-propelled by water-restricted mice, and the movement speed was measured by a rotary encoder using Arduino-based microcontrollers. The digitized speed signal interfaced with a behavior control system (Bpod r0.9–1.0, Sanworks) via custom MATLAB code (2019b, MathWorks) on a Windows PC. Reward delivery (10% sucrose water) was controlled by a solenoid valve (The Lee Co.) through a custom-made lick port, with licking detected by a custom-fabricated optical sensor (FX-300 series, Panasonic). The Bpod system interfaced with the rotary encoder, solenoid valve and lick sensor. Behavior data were digitized through a PCIe-6343 X-series DAQ system (National Instruments) running WaveSurfer software (v.0.982, Janelia).

After ≥6 days of recovery from optical window surgery, mice were placed on water restriction (1.5 ml day^−1^). Animals were then handled by the experimenter daily (10–30 min) for at least ≥4 days (typically 5–6 days). We then introduced the treadmill system to the head-fixed mice to run on a tactile-cue-featured belt, receiving 10% sucrose water at epoch-dependent increasing distances (8–15 laps per epoch). Reward distances were fixed within epochs but increased between epochs. Manual rewards were initially provided to encourage running. Once the reward distance reached 126 cm, the reward was delivered at a fixed location (50 cm) on the belt for ≥4 days (typically 6 days, ~700 laps) before beginning the reward-switch experiment. On ‘day 0’, this experiment concluded, and the reward switch began on ‘day 1’: rewards were delivered at 50 cm for the first 100 laps, followed by 140 cm for the next 100 laps. Individual sessions typically lasted 30–60 min, with one session per day. Two belts were used: belt A was uniformly covered with three cues (Velcro, glue stick and white fabric; 60 cm per cue zone) and belt B featured six distinct cues (30 cm per cue zone).

### Two-photon imaging

All two-photon Ca^2+^ imaging recordings were performed in the dark using a custom-made microscope (Janelia MIMMS 2.0). Transgenically expressed GCaMP6f in the hippocampal CA1 was excited at 920 nm (typically 30–50 mW, measured at the objective) by a Ti:Sapphire laser (Chameleon Ultra II, Coherent) and imaged through a Nikon ×16, 0.8 numerical aperture objective. The emission light passed through a 565 DCXR dichroic filter (Chroma) and a 531/46 nm bandpass filter (Semrock) and was detected by a GaAsP photomultiplier tube (11706P-40SEL, Hamamatsu). Images (512 × 512 pixels) were acquired at ~30 Hz using ScanImage software (Vidrio Technologies).

Imaging fields of view (FOVs) (~300 × 300 µm) were selected based on visible Ca^2+^ transients in the somata. For longitudinal imaging, a reference FOV was first chosen and then registered daily. Experiments were terminated if substantial differences were observed on subsequent days in the FOV.

### In vivo intracellular recordings

The intracellular recordings were performed as previously described^61,62^. Briefly, an extracellular local field potential electrode was first lowered into the dorsal hippocampus using a micromanipulator until prominent theta-modulated spiking and increased ripple amplitude were detected after passing through the neocortex, usually at a depth of 1.0–1.2 mm. A glass intracellular recording pipette was then lowered to the same depth while applying positive pressure (~9.5 psi). The intracellular solution contained 134 mM K-gluconate, 6 mM KCl, 10 mM HEPES, 4 mM NaCl, 0.3 mM MgGTP, 4 mM MgATP, 14 mM tris-phosphocreatine and 0.2% biocytin. Current-clamp recordings of intracellular membrane potential (*V*_m_) were amplified and digitized at 20 kHz without correction for the liquid junction potential.

### Data analysis

#### Pre-processing of imaging data and signal processing

Acquired two-photon images were motion-corrected using Suite2p^80^ (Python version; http://github.com/MouseLand/suite2p). For longitudinal imaging across multiple days, data acquired each day were concatenated and motion-corrected. Regions of interest (ROIs) were automatically selected and time series fluorescence traces were extracted using Suite2p. Manual curation discarded unwanted ROIs and reselected ROIs rejected by Suite2p based on the anatomy in the Suite2p graphical user interface. ROI contamination was examined manually: ~10–20 nearby ROIs were selected, and the time series fluorescence traces were visually inspected to identify ‘ROI-x contaminates ROI-y’ pairs. Two rounds of inspections were performed for each FOV discarding ~40% ROIs. No neuropil subtraction was applied. Further analyses were conducted using custom code in MATLAB (v.2021a). Briefly, the raw fluorescence signals were baseline-corrected (5,000-frame window) before being converted to Δ*f*/*f* ((*F* – *F*_0_)/*F*_0_), where *F*_0_ was the histogram mode of *F*. Notable calcium transients exceeded 3 × standard deviation above the baseline noise, which was estimated from deviations below peak Δ*f*/*f* histogram values. Spatial maps of Δ*f*/*f* for each ROI were then generated using frames where the animal’s velocity exceeded 5 cm s^−1^. The ~180-cm belt was uniformly divided into 50 spatial bins (3.6 cm per bin) and the mean Δ*f*/*f* was calculated per bin. Gaussian smoothing (three bins) was applied to each Δ*f*/*f* map solely for display purposes. To ensure consistent lap counts, only the first 100 laps before and after the reward switch were analyzed. The reward was located at bin 14 (50 cm) before the switch and at bin 39 (140 cm) after. All day 0 sessions had ≥100 laps. If fewer than 100 laps were available, all available laps were analyzed. Reward positions were indicated in all figures.

### Place-cell identification

We identified the onset lap of each cell as the first lap where the current lap and ≥2 out of the next five laps had ≥3 consecutive spatial bins with significant events (>3 × s.d. above the baseline), detected within 90 cm (±45 cm) around the PF peak location. PFs were defined as the trial-averaged Δ*f*/*f* activity. The maximum, but not the mean, Δ*f*/*f* per bin determined notable events. Only the first onset lap per condition (for example, before versus after the reward switch) was selected. Trials subsequent to the onset lap determined if a cell was a PC.

A cell had spatially modulated activity if its PF provided sufficient spatial information (SI) about the linear track (>95th percentile of the shuffled SI). The SI for each cell was calculated as previously described^31^:

$${\rm{SI}}=\sum _{i}{p}_{i}\times {x}_{i}\times{\log }_{2}({\rm{abs}}(x_{i}/\bar{x}))$$

where *p*_*i*_ is the occupancy probability for bin *i*, *x*_*i*_ is the smoothed mean activity in bin *i*, and $\bar{x}$ is the overall mean activity. SI was then compared to 200 shuffled activity datasets. For each shuffle, we circularly shifted the Δ*f*/*f* time series traces by ≥500 frames and further divided into six trunks with randomly permuted orders. Cells with observed SI exceeding the 95th percentile of the shuffled datasets were considered spatially modulated. A cell was considered reliable if >20% of the post-onset laps had notable events. PCs were both spatially modulated and reliable. Onset was set to ‘NaN’ if not identified, and PC identification continued from lap 1. No additional processing was performed for multiple PFs.

### Spatial map of the behavioral data

Spatial maps of running velocity and lick counts were generated by dividing the 180 cm belt into 50 bins (3.6 cm per bin). Mean velocity (cm s^−1^) and total lick count were calculated per bin, discarding data with velocity <5 cm s^−1^. Licking probability was calculated as the fraction of laps with ≥1 lick per bin.

### Identification of running starts after reward

Post-reward stop time was identified within 5 s or 50 cm of travel as the animal’s speed fell below 1 cm s^−1^ (or 5 cm s^−1^ if absent, or the lowest speed if both were absent). Next, the cumulative distance from reward was zeroed at the stop time. A stationary period was defined as the animal’s speed <1 cm s^−1^ for ≥0.5 s, from the reward to 0.18 cm post-stop. The end of the stationary period was determined through a 1-s smoothing filter (>1 cm s^−1^ was set as 1, <1 cm s^−1^ was set as 0). Small running bouts (<8 cm) were removed, and the speed was reset to zero only for finding consistent running starts. This method effectively identified stationary periods in most laps. Manual determination was used for ~5% of unclear cases. Our analysis included only the first 100 laps before the reward switch on day 1.

### Identification of non-rewarded running starts

Time series were resampled at ~6.7 Hz (0.15 s per point). Non-rewarded running starts were automatically identified as the end of a stationary period (velocity <1 cm s^−1^ for ≥1 s), ≥10 cm from the previous reward location. The first post-reward running start was excluded. This analysis only included 100 laps before the reward switch.

### Determination of place-cell types

We defined PCs as ‘space’ (spatial cue-referenced) that maintained stable PFs relative to spatial cues on the belt after the reward switch (0–15 cm); ‘goal’ (reward goal-referenced) that shifted PFs following the reward (75–90 cm shifts); and ‘inter’ (intermediate) PCs that shifted 16–74 cm. Extended Data Fig. 1j contains additional details.

### Population vector correlation analysis

To understand how the PC population encodes location, we performed PV analysis^81^. PVs are activity vectors in which each element represents a neuron’s activity at a given location. The Pearson correlation between PVs across all locations produces the similarity matrix *M*. Each element *M*_*i*,*j*_ represents the correlation coefficient between PVs at location *i* and location *j*. *M* has dimensions of 50 by 50 (50 bins per track), measuring the position representation similarity between conditions. When comparing PVs before versus after the reward switch, we considered four scenarios: (1) If the PC representation was space-referenced and unaffected by the reward, we expected high similarity along the diagonal. (2) If the PC representation was goal-referenced that exclusively encoded the reward (run start) distance, we expected high similarity along the off-diagonal (a 90 cm shift). (3) If space and goal PCs had location preferences (for example, goal PCs predominantly clustered after the reward, space PCs elsewhere), we expected fragmented high-similarity patches aligned to the diagonal (space-referenced) and off-diagonal (goal-referenced). (4) If Intermediate PCs dominated and their shared PF locations shifted similar distances, we expected high similarity between the diagonal and off-diagonal. To quantitatively compare space- versus goal-referenced spatial coding, we averaged diagonal and off-diagonal elements of *M* for ‘space similarity’ and ‘goal similarity’, respectively.

### Space versus time analysis

We adapted a previously published space-time analysis for our calcium imaging data^55^. Each lap started from the post-reward running onset and ended at the next lap’s stop after reward. To generate the space and time maps for each cell, we uniformly divided the distance and duration of each lap into 50 bins and averaged the Δ*f*/*f* within each bin. Lap distances were approximately 180 cm, with minor variations. The lap duration was determined from the median full-lap time of 100 laps before the reward switch. Therefore, the lap-by-lap duration was fixed within animals but varied between animals. We then compared space and time coding for goal PCs by categorizing 100 laps into ‘slow,’ ‘medium’ and ‘fast’ based on the mean running speeds from lap start to the PF peak location in space. PCs with peak positions over 90 cm from the running onset or with substantial peak position discrepancies between the slowest and fastest laps were excluded, focusing on cells near the reward or running onset.

Two scenarios were considered:

1. Spatially stable PCs: maintained consistent spatial peak positions across varying speeds but peaked later in time during slower laps.
2. Temporally stable time cells: maintained consistent temporal peak positions across varying speeds but peaked earlier in space during slower laps.

### BTSP event identification

We adapted a previously published BTSP analysis^48,70,82^ with some modifications. Putative BTSP events were identified as (1) a ‘strong’ notable Ca^2+^ event with an amplitude in the top tenth percentile of all Ca^2+^ events in the same session and cell. Separate thresholds were used for laps before and after the reward switch. (2) Only laps with peak Δ*f*/*f* within ~45 cm of the BTSP event peak were considered. (3) Four out of five subsequent laps had notable Ca^2+^ events. (4) Δ*f*/*f* amplitude of the following five laps increased >100% compared to the preceding five laps. (5) The peak Δ*f*/*f* of BTSP events was >2.

We did not include BTSP events during the last five laps (laps 96–100, laps 196–200) due to a lack of sampling laps, and relaxing this criterion did not change our main conclusions. For identified notable Ca^2+^ events, the PF was quantified as consecutive spatial bins where max Δ*f*/*f* exceeded 35% of the peak of the max Δf/f value. The total number of such bins was the PF width. The PF of the mean subsequent or preceding five-lap activity was defined as^83^ (1) consecutive spatial bins where max Δ*f*/*f* exceeded 35% of the peak of max Δ*f*/*f* values; (2) PF width <90% of the track; (3) in-field max Δ*f*/*f* > 0.2; and (4) mean in-field Δ*f*/*f* > 2 times that of out-field. The COM of BTSP events was calculated using:

$${\rm{COM}}_{n}=\frac{\sum _{i}({x}_{i}{\rm{ \times}}{d}_{i})}{\sum _{i}{x}_{i}}$$

where *x*_*i*_ is the max Δ*f*/*f* within spatial bin *i*, and *d*_*i*_ is the spatial position. The COM calculation used only within-PF spatial bin data. If no PFs were found for the mean activity of the subsequent or preceding five laps, the COM was set to ‘NaN’. The induction velocity was defined as the mean velocity within the identified PF.

### Intracellular recordings

Acquired intracellular recording data were analyzed using custom-written code in either MATLAB (v.2019a) or IGOR (v.8.04). Spatial action potential (AP) rates were calculated as the number of APs divided by the time spent within 1-cm spatial bins, averaged across trials. For *V*_m_ ramps, APs were removed, and baseline correction was applied by subtracting the difference between the AP threshold (the most negative fifth percentile) and –50 mV on a trial-by-trial basis. The corrected data were then spatially binned and averaged as described above. Heatmaps of spatially binned AP rates and *V*_m_ ramps were smoothed solely for display using a Gaussian filter with 5 bins (5 cm) and 11 bins (11 cm), respectively. The PF location was defined as the COM of the smoothed mean AP rate, excluding bins with firing rates <10% of the peak, across all trials before/after the reward switch (or before/after plateaus as stated in the figures or text). The goal PF was set as 92 cm away from the original (space) PF before the reward switch.

### Goal *V*_m_, space *V*_m_ and goal/space index

To calculate the putative goal *V*_m_ ramps, *V*_m_ ramps were first aligned to the physical track locations (0 cm on the belt). *V*_m_ ramps from all the trials before (after plateaus, if any; ≥4 trials, 15.08 ± 0.63 trials) and after (before plateaus, if any; ≥3 trials, 19.88 ± 2.48 trials) the reward switch were averaged. The difference between post- and pre-reward switch *V*_m_ ramps (Δ*V*_m_ = *V*_m_post_ – *V*_m_pre_) was computed. The goal *V*_m_ was defined as the area under the curve (AUC) of Δ*V*_m_ ramps within ±30 cm around the goal PF (92 cm from the original PF; red window in Extended Data Fig. 7e). Space *V*_m_ ramps were calculated similarly but were referenced to the start of running before subtraction. The space-referenced PF shifted 92 cm from the original PF when aligned to the running start, and the space-referenced area (±30 cm around the space-referenced PF, blue window in Extended Data Fig. 7i) was used to compute the space *V*_m_ (AUC of this area). The goal/space index for each cell was then calculated as:

$${\rm{Index}}=\frac{{{\rm{Area}}}_{{\rm{Goal}}}-{{\rm{Area}}}_{{\rm{Space}}}}{{{\rm{Area}}}_{{\rm{Goal}}}+{{\rm{Area}}}_{{\rm{Space}}}}$$

One cell was excluded due to immediate, spontaneous plateau potentials following the reward switch. The symmetry of Δ*V*_m_ for each cell is defined as:

$$\begin{array}{l}{\rm{Symmetry}}=\\\displaystyle\frac{\min \left({{\rm{Area}}}_{{\rm{Goal}}\_{\rm{left}}},{{\rm{Area}}}_{{\rm{Goal}}\_{\rm{right}}}\right)+\min \left({{\rm{Area}}}_{{\rm{Space}}\_{\rm{left}}}{,{\rm{Area}}}_{{\rm{Space}}\_{\rm{right}}}\right)}{\max \left({{\rm{Area}}}_{{\rm{Goal}}\_{\rm{left}}},{{\rm{Area}}}_{{\rm{Goal}}\_{\rm{right}}}\right)+\max \left({{\rm{Area}}}_{{\rm{Space}}\_{\rm{left}}},{{\rm{Area}}}_{{\rm{Space}}\_{\rm{right}}}\right)}\end{array}$$

The left and right areas were defined as –30 to 0 cm and from 0 to 30 cm relative to the goal- or space-referenced PF, respectively. An additional nine cells were excluded due to spontaneous plateau potentials (four cells, marked with ‘*’ in Extended Data Fig. 9a) or the emergence of two distinct PFs after the reward switch (five cells, marked with ‘#’ in Extended Data Fig. 9a). PF *V*_m_ ramps were reconstructed by summing the goal *V*_m_ and space *V*_m_ for each cell.

The calculation of goal *V*_m_ and space *V*_m_ has limitations and should be considered approximate. These values are derived by subtracting a baseline (*V*_m_ before the reward switch), which could underestimate depolarization at the goal or space PF location if baseline activity is present; however, as all PFs were induced by repetitive plateau initiation at the beginning of each recording, baseline depolarizations were generally small, resulting in minimal errors. Broad fields (>90 cm) may also lead to narrow Δ*V*_m_ peaks flanked by broad negatives on both sides (for example, Extended Data Fig. 8a, the third and eighth cell in the space PC), underestimating both goal *V*_m_ and space *V*_m_ without substantially affecting their ratio. To reduce overlap errors, we restricted the goal and space areas to ±30 cm rather than ±45 cm around their respective PFs. Overall, the reconstructed PF *V*_m_ ramps aligned well with the original PF *V*_m_ ramps, particularly in the PF center, supporting the method’s accuracy.

### Plateau potentials

Spontaneous long-lasting plateau events were defined as plateau potentials (*V*_m_ > −35 mV) lasting over 100 ms, with inter-plateau intervals >10 ms. For PF position analysis before and after a plateau, only plateaus with at least two plateau-free trials both preceding and following the event were included.

### Statistical methods

The exact sample size (*n*) for each experimental group is reported in the figure legends or main text. While no statistical tests were used to predetermine sample sizes, our sample sizes align with previous publications (population imaging^47,82,84,85^ and in vivo whole-cell^62,65,66^) that used similar behavior tasks and were guided by the number of neurons that could be imaged or patched in awake, behaving mice. In some cases, where data distribution was assumed but not formally tested, parametric *t*-tests were applied to analyze the data. Experiments were randomized by randomly assigning littermate mice to the experimental groups. Data analyses were performed automatically without consideration of trial types or experimental groups, but they were not blinded to the experimenter. Unless otherwise indicated in the figures, data were presented as mean ± s.e.m.

### Reporting summary

Further information on research design is available in the Nature Portfolio Reporting Summary linked to this article.

## Online content

Any methods, additional references, Nature Portfolio reporting summaries, source data, extended data, supplementary information, acknowledgements, peer review information; details of author contributions and competing interests; and statements of data and code availability are available at 10.1038/s41593-025-01930-5.

## Supplementary information

Reporting Summary

## Source data

Source Data Fig. 1 Statistical source data.

Source Data Fig. 1 Source image for Fig. 1a, middle.

Source Data Fig. 2 Statistical source data.

Source Data Fig. 3 Statistical source data.

Source Data Fig. 4 Statistical source data.

Source Data Fig. 5 Statistical source data.

Source Data Extended Data Fig. 1 CA1 PC representation before and after a reward switch.

Source Data Extended Data Fig. 2 Intermediate PCs from day 1 on a familiar versus novel belt.

Source Data Extended Data Fig. 3 Goal PCs encoded reliable distance but not time information after the running start and did not require reward per se.

Source Data Extended Data Fig. 4 Experience-dependent, flexible space- and goal-referenced CA1 PC representation.

Source Data Extended Data Fig. 5 Longitudinal tracking of cells from day 1 to day 2.

Source Data Extended Data Fig. 6 Additional characteristics of CA1 PC remapping.

Source Data Extended Data Fig. 7 Calculating the putative goal- and space-referenced *V*_m_ by subtraction.

Source Data Extended Data Fig. 8 The reconstructed PF *V*_m_ and original PF *V*_m_ in individual CA1 PCs.

Source Data Extended Data Fig. 9 Putative goal- and space-referenced *V*_m_ in individual CA1 PCs.

Source Data Extended Data Fig. 10 Spontaneous plateau potential initiation after the reward switch.

## Data Availability

The data supporting this study’s findings are available from the corresponding author upon request. Source data are provided with this paper.

## Code availability

Scripts that support the results of this study are available on GitHub repositories at https://github.com/FishQian/CA12025Paper_SpaceGoalReferencing.
