# Supplementary material for: Mechanisms of experience-dependent place-cell referencing in hippocampal area CA1
Source: Nat Neurosci. 2025 Apr 1;28(7):1486–96. doi: 10.1038/s41593-025-01930-5 (PMC12229891; doi:10.1038/s41593-025-01930-5)
Supplement: Supplementary file 1 — Reporting Summary [file 41593_2025_1930_MOESM1_ESM.pdf]

nature portfolio

Corresponding author(s): Jeffrey C. Magee

Last updated by author(s): Jan 24, 2025

Reporting Summary

Nature Portfolio wishes to improve the reproducibility of the work that we publish. This form provides structure for consistency and transparency in reporting. For further information on Nature Portfolio policies, see our Editorial Policies and the Editorial Policy Checklist.

Statistics

For all statistical analyses, confirm that the following items are present in the figure legend, table legend, main text, or Methods section.

n/a	Confirmed	
☐	☒	The exact sample size (*n*) for each experimental group/condition, given as a discrete number and unit of measurement
☐	☒	A statement on whether measurements were taken from distinct samples or whether the same sample was measured repeatedly
☐	☒	The statistical test(s) used AND whether they are one- or two-sided<br>*Only common tests should be described solely by name; describe more complex techniques in the Methods section.*
☐	☒	A description of all covariates tested
☐	☒	A description of any assumptions or corrections, such as tests of normality and adjustment for multiple comparisons
☐	☒	A full description of the statistical parameters including central tendency (e.g. means) or other basic estimates (e.g. regression coefficient) AND variation (e.g. standard deviation) or associated estimates of uncertainty (e.g. confidence intervals)
☐	☒	For null hypothesis testing, the test statistic (e.g. *F*, *t*, *r*) with confidence intervals, effect sizes, degrees of freedom and *P* value noted<br>*Give P values as exact values whenever suitable.*
☒	☐	For Bayesian analysis, information on the choice of priors and Markov chain Monte Carlo settings
☒	☐	For hierarchical and complex designs, identification of the appropriate level for tests and full reporting of outcomes
☐	☒	Estimates of effect sizes (e.g. Cohen's *d*, Pearson's *r*), indicating how they were calculated

Our web collection on statistics for biologists contains articles on many of the points above.

Software and code

Policy information about availability of computer code

Data collection	The Ca2+ imaging data were recorded using a National Instruments PXI system controlled by ScanImage (R2021, Vidrio). The behavioral data were acquired using an NI PCIe-6343 card connected to a BNC-2090A rack-mounted breakout box, controlled by a Bpod module (r0.9–1.0, Sanworks) using custom MATLAB code (2019b, MathWorks) running on a Windows PC. The animal's licking was detected by a custom-fabricated optical lickometer (FX300-series, Panasonic). Behavioral data were digitized through a PCIe-6343 X-series DAQ system (National Instruments) and saved via WaveSurfer software (v0.982, Janelia, open-access, https://github.com/JaneliaSciComp/Wavesurfer).<br><br>The intracellular recordings were performed in current-clamp mode using a Dagan BVC-700A amplifier. Both recording and behavioral data were digitized at 20 kHz using an NI PCIe-6343 card, connected to BNC-2090A breakout box, controlled by the open-source software Wavesurfer (v0.982, Janelia).
Data analysis	Acquired two-photon Ca2+ images of CA1 somata were motion-corrected using Suite2p (v0.10.2, Python version, http://github.com/MouseLand/suite2p). Regions of interest (ROIs) were selected, and time-series fluorescence traces were extracted automatically using Suite2p. Further analyses were performed using custom-written functions in MATLAB (v2021a). Mean field-of-view images was acquired using Fiji's Z-project function (open-access software, macOS v2.14.0/1.54f, https://fiji.sc/).<br>Acquired intracellular recording data were further analyzed using custom-written code in either MATLAB (v2019a) or IGOR (v8.04).<br><br>Note: The MATLAB code to analyze our datasets is available via: https://github.com/FishQian/CA12025Paper_SpaceGoalReferencing

For manuscripts utilizing custom algorithms or software that are central to the research but not yet described in published literature, software must be made available to editors and reviewers. We strongly encourage code deposition in a community repository (e.g. GitHub). See the Nature Portfolio guidelines for submitting code & software for further information.

Data

Policy information about availability of data

All manuscripts must include a data availability statement. This statement should provide the following information, where applicable:

- Accession codes, unique identifiers, or web links for publicly available datasets
- A description of any restrictions on data availability
- For clinical datasets or third party data, please ensure that the statement adheres to our policy

The data that support the current study are available from the corresponding author upon reasonable request.

Research involving human participants, their data, or biological material

Policy information about studies with human participants or human data. See also policy information about sex, gender (identity/presentation), and sexual orientation and race, ethnicity and racism.

Reporting on sex and gender	N/A
Reporting on race, ethnicity, or other socially relevant groupings	N/A
Population characteristics	N/A
Recruitment	N/A
Ethics oversight	N/A

Note that full information on the approval of the study protocol must also be provided in the manuscript.

Field-specific reporting

Please select the one below that is the best fit for your research. If you are not sure, read the appropriate sections before making your selection.

☒ Life sciences ☐ Behavioural & social sciences ☐ Ecological, evolutionary & environmental sciences

For a reference copy of the document with all sections, see nature.com/documents/nr-reporting-summary-flat.pdf

Life sciences study design

All studies must disclose on these points even when the disclosure is negative.

Sample size	No statistical tests were used to predetermine sample sizes, but our sample sizes are similar to those reported in previous publications (population imaging: refs. 47, 82, 84, 85); and in vivo whole-cell: refs. 62, 65, 66; see also Zhao et al., Neuron 2022) using a similar behavioral task and were guided by the number of neurons that could be imaged using two-photon microscopy or patched in awake, behaving mice. The main effects were significant with the number of mice, neurons in each group, and the effects were consistent across individual mice and neurons within each group, as evident by the presentation of individual data points throughout the paper.
Data exclusions	Ca2+ imaging: Animals were excluded from further analyses for two reasons: 1) extensive z-motion that precluded imaging of the same population of neurons throughout the recording session; 2) during the reward switch experiment, animals did not switch behavior to lick and stop at the new locations which precluded testing the distinct roles of different reference frames<br>Intracellular recordings: Recording data were excluded if the recording time was not long enough to cover the reward switch experiment. One cell was excluded from Goal/Space index analysis because of immediate, spontaneous plateau potentials after the reward switch. Another 9 cells were excluded from the symmetry/reconstruction analysis because of either spontaneous plateau potentials that switched the category of place cells (4 cells) or the appearance of two separate place fields after the reward switch (5 cells).
Replication	We used appropriate sample sizes and indicated them throughout the manuscript. All experiments were performed independently, and we explicitly reported the number of replications for each experiment in the text. For instance, imaging experiments were performed independently across 5–6 animals in Figures 1, 2, 3, and 5. Intracellular recording experiments were performed independently across 16 animals in Figure 4 (26 recorded cells). In most analyses, we provided individual data points representing individual animals to demonstrate consistency across experiments. For plots with too many bins making visualization difficult, summary statistics were shown. All experiments were successfully replicated, and the findings were consistent across all replications.
Randomization	Ca2+ imaging data: Littler mate GP5.17 mice were used and randomly assigned to each experiment. We compared the experimental group to the appropriate control group for all experiments.<br>Intracellular data: Only wild-type male C57BL/6 mice were used for all recordings. For most cases, the comparison was performed within each mouse between trials before and after the reward switch. This is consistent with previous studies using similar techniques (refs. 62, 65, 66; see also Zhao et al., Neuron 2022).

Blinding	Experiments and data analyses were not performed blind to the experimental conditions. This was because the experimenter applied different belts, or performed the surgeries and was thus not able to be blind to the experimental conditions. However, all analyses were performed using automated data analyses procedures without consideration of trial types or experimental groups.

Reporting for specific materials, systems and methods

We require information from authors about some types of materials, experimental systems and methods used in many studies. Here, indicate whether each material, system or method listed is relevant to your study. If you are not sure if a list item applies to your research, read the appropriate section before selecting a response.

Materials & experimental systems

n/a	Involved in the study	
☒	☐	Antibodies
☒	☐	Eukaryotic cell lines
☒	☐	Palaeontology and archaeology
☐	☒	Animals and other organisms
☒	☐	Clinical data
☒	☐	Dual use research of concern
☒	☐	Plants

Methods

n/a	Involved in the study	
☒	☐	ChIP-seq
☒	☐	Flow cytometry
☒	☐	MRI-based neuroimaging

Animals and other research organisms

Policy information about studies involving animals; ARRIVE guidelines recommended for reporting animal research, and Sex and Gender in Research

Laboratory animals	All experiments were performed in adult GP5.17(ref. 77; n = 22 mice, at least 8 weeks old on the day of surgery) of either sex or WT male C57BL/6 (n = 16 mice, 8–12 weeks old). The experimenter was not blind to the different conditions of the experiments. Animals were housed in the Magee Satellite under an inverse 12-hour dark/12-hour light cycle (light time: 9 pm–9 am), with controlled temperature (around 21°C) and humidity (around 30–60 %).
Wild animals	No wild animals were used in the study
Reporting on sex	Ca2+ imaging: Animals from either sex were randomly used for our experimental groups.<br>Intracellular recordings: Only male mice were used.
Field-collected samples	No field-collected samples were used in the study
Ethics oversight	All experimental procedures performed were approved by the Baylor College of Medicine Institutional Animal Care and Use Committee (Protocol AN-7734).

Note that full information on the approval of the study protocol must also be provided in the manuscript.

Plants

Seed stocks	N/A
Novel plant genotypes	N/A
Authentication	N/A
