# Supplementary figures and images for: Mechanisms of experience-dependent place-cell referencing in hippocampal area CA1
Source: Nat Neurosci. 2025 Apr 1;28(7):1486–96. doi: 10.1038/s41593-025-01930-5 (PMC12229891; doi:10.1038/s41593-025-01930-5)

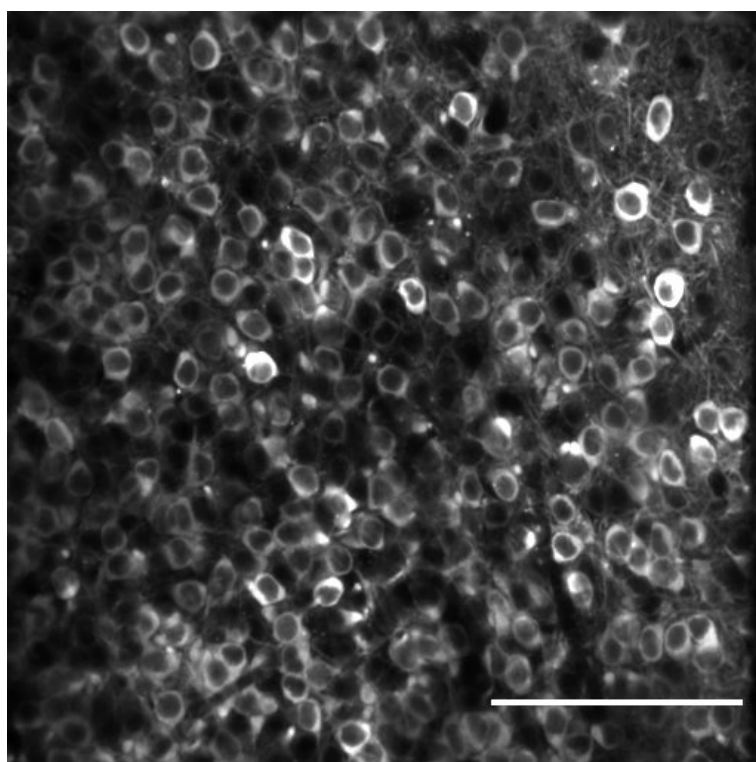

Supplement: Supplementary file 3 — Source image for Fig. 1a, middle. [file 41593_2025_1930_MOESM3_ESM.pdf]
